# Isolation, Detection and Analysis of Circulating Tumour Cells: A Nanotechnological Bioscope

**DOI:** 10.3390/pharmaceutics15010280

**Published:** 2023-01-13

**Authors:** Upama Das, Soumyabrata Banik, Sharmila Sajankila Nadumane, Shweta Chakrabarti, Dharshini Gopal, Shama Prasada Kabekkodu, Pornsak Srisungsitthisunti, Nirmal Mazumder, Rajib Biswas

**Affiliations:** 1 Applied Optics and Photonics Laboratory, Department of Physics, Tezpur University, Tezpur 784028, Assam, India; 2 Department of Biophysics, Manipal School of Life Sciences, Manipal Academy of Higher Education, Manipal 576104, Karnataka, India; 3 Department of Bioinformatics, Manipal School of Life Sciences, Manipal Academy of Higher Education, Manipal 576104, Karnataka, India; 4 Department of Cell and Molecular Biotechnology, Manipal School of Life Sciences, Manipal Academy of Higher Education, Manipal 576104, Karnataka, India; 5 Department of Production and Robotics Engineering, Faculty of Engineering, King Mongkut’s University of Technology North Bangkok, Bangkok 10800, Thailand

**Keywords:** circulating tumour cells, metastasis, nanotechnology, nanomaterials, isolation, detection, cancer

## Abstract

Cancer is one of the dreaded diseases to which a sizeable proportion of the population succumbs every year. Despite the tremendous growth of the health sector, spanning diagnostics to treatment, early diagnosis is still in its infancy. In this regard, circulating tumour cells (CTCs) have of late grabbed the attention of researchers in the detection of metastasis and there has been a huge surge in the surrounding research activities. Acting as a biomarker, CTCs prove beneficial in a variety of aspects. Nanomaterial-based strategies have been devised to have a tremendous impact on the early and rapid examination of tumor cells. This review provides a panoramic overview of the different nanotechnological methodologies employed along with the pharmaceutical purview of cancer. Initiating from fundamentals, the recent nanotechnological developments toward the detection, isolation, and analysis of CTCs are comprehensively delineated. The review also includes state-of-the-art implementations of nanotechnological advances in the enumeration of CTCs, along with future challenges and recommendations thereof.

## 1. Introduction

**Figure 1:**
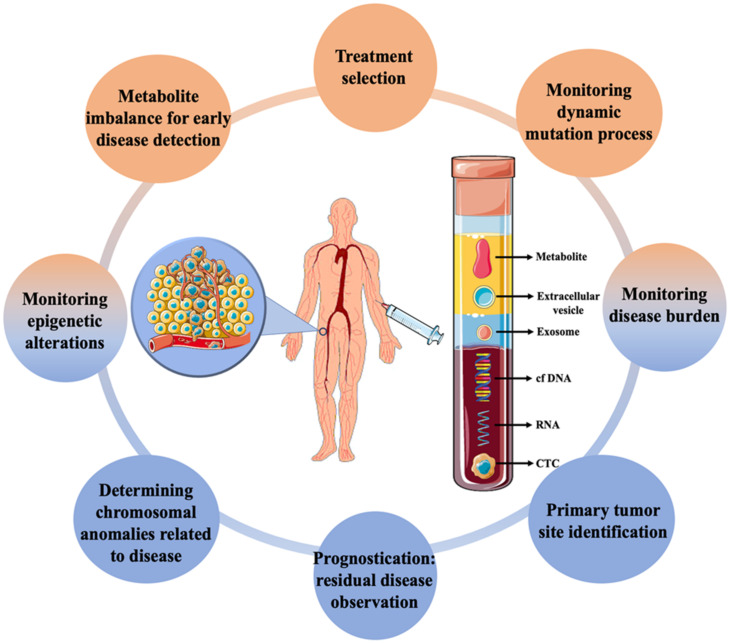
Applications of liquid biopsy in cancer diagnosis along with the different biomarkers commonly targeted using the technique.

Cancer is a broad term that often refers to a set of diseases characterized by uncontrolled cell growth with the ability to spread throughout the animal body [1]. It is one of the major causes of mortality globally, affecting patients from all socioeconomic backgrounds and their families. According to World Health Organization (WHO) statistics, 18.1 million new cases and 9.6 million deaths occurred in 2018 due to cancer, with secondary cancer or metastasis accounting for more than 90% of them [2,3]. The growing prevalence of cancer has encouraged the development of rapid and sensitive techniques for early detection. Currently, several methods, such as detecting the levels of metabolites or tumour markers in the blood, biopsy followed by histological evaluation, ultrasonography, magnetic resonance imaging, computerised tomography, positron emission tomography, and endoscopic-based examination are available [4]. The majority of the techniques are routinely used for cancer diagnosis and disease monitoring at a later stage only after the appearance of initial symptoms. As cellular and molecular sciences advance, various indicators and markers for early cancer detection are being investigated. Biomarkers based on gene and RNA expression are regularly used for the expeditious evaluation of cancers [5,6,7]. However, the requirements of extensive facilities and expensive instruments have limited its diagnostic application. On the other hand, dissemination of circulating tumour cells (CTCs) from primary tumours due to metastasis—which then spreads into the lymphatic nodes and blood, has been seen as an important marker for early diagnosis of cancer [8]. They stay either as a single cell or in a group, which can move from one site to another causing tumour [9] The early invasion of CTCs along with fast progression is vital for accurate monitoring, and detection of the tumour cells and an important part of liquid biopsy [10,11]. Detection and genomic characterization of CTCs can also help to investigate the mechanism of metastasis [12,13]. Liquid biopsy, often referred to as fluid biopsy or liquid phase biopsy, is a non-invasive diagnostic method for the analysis of non-solid biological samples such as blood. It overcomes the limitation of the inability to obtain heterogeneous information with traditional methods and helps in the identification of both genetic and epigenetic causes related to any diseases [14]. Liquid biopsy is not a routine test in clinical practice; however, over the past few years, it has evolved as a promising tool for the effective and early detection, monitoring of recurrence and evaluation treatment efficacy of cancer. The early description of circulating free DNA (cf DNA) and RNA in 1948, without knowing in detail about it, is said to be the first step towards the biopsy [15]. Ever since, various other biomarkers have also been looked into, for effective disease management (Figure 1). Micro-RNA, cf DNA, circulating tumour DNA, metabolites, CTCs, exosomes and other extracellular vesicles are some of the markers which have been significantly worked upon. Researches isolate these markers from blood and analyze them by different biochemical and analytical methods such as next-generation sequencing, polymerase chain reactions and mass spectrometry-based genotyping assay [7,16]. With the advancement of cancer treatment towards a shift to personalized medicine, this developing technology has the potential to change and/or complement functional cancer care. The advancement of liquid biopsy procedures has resulted in the incorporation of extremely effective approaches for the rapid detection and separation of CTCs [17], with nanotechnology at the forefront. Due to the unique physicochemical properties of nanomaterials emerging from their high surface area, shape, size, and optical nature, nanotechnology has proven to be the most reliable strategy in cancer diagnosis [18]. Higher surface-to-volume ratio property allows strong binding of the ligands which helps in recognizing critical biomarker molecules. As such, with the advent of this strategy, CTCs isolation and detection with higher efficiency and specificity, even at extremely low numbers in the circulatory system, has recently been achievable, allowing for early cancer diagnosis [19].

It is pertinent to mention that research activity in this direction has risen to a large extent. There are a number of reports showcasing innovative implementations of probing and targeting CTCs. On a closer look, the abundant literature reports about isolation and detection implementing nanotechnology were found separately. Notwithstanding, few articles reported about these methods compared them in the same way, and with illustrative examples. As such, it is no exaggeration to say that there is a dearth of exhaustive articles which can provide readers with a panoramic review of the recent development in nanotechnology-based strategies for isolation, detection and targeting of CTCs. Starting from basics and principles, we provide an exhaustive summary of CTCs and their biology along with inputs on nanosystems for tumour cell evaluation. Moreover, the technologies involved in the isolation, probing and analysis of CTCs using different nanomaterials have been described extensively. This work provides a guiding point to the reader, to facilitate understanding of current nanotechnology platforms which are changing traditional approaches for the diagnosis of cancer.

## 2. Biogenesis of CTCs

**Figure 2:**
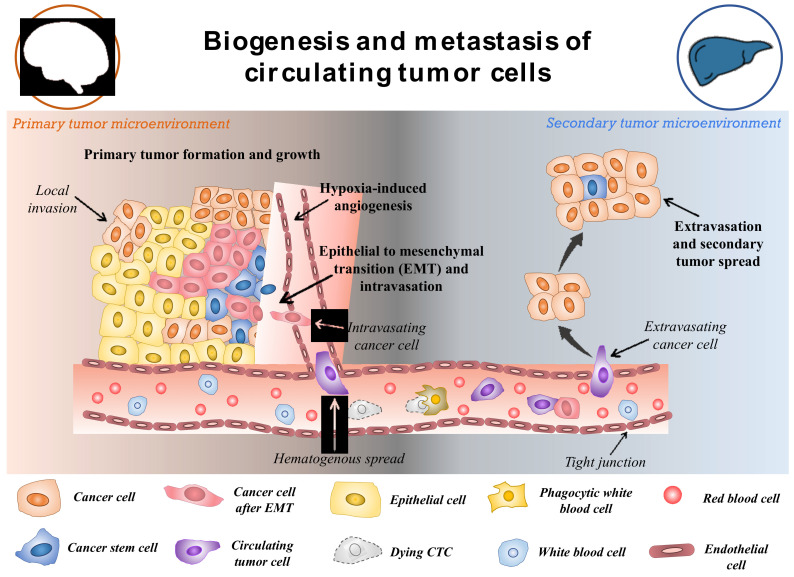
Schematic representation of potential biogenesis and the transport mechanism of circulating tumor cells. Reproduced from [38].

CTCs are a rare subset of cells that are released from primary tumours and act as the precursor for the growth of additional cancerous tissue in a different location in the body [20]. The hallmarks of cancer such as invasiveness and motility give rise to the ability of malignant tumours to be metastatic. This is devised to be the main factor for the formation and release of CTCs in the circulatory system [21]. Though the formation and progression of cancer is a complex and little understood process, with the development of modern tools, this can be explored. The metastasis and carcinogenesis of tumour cells are commonly described to be a multi-step process, as shown in Figure 2 [22]. Initially, the cell loses its normal cell cycle ability, whether being triggered by internal factors or due to extremal stimulus. This causes the tumour cells to accumulate numerous mutations, prompting them to become malignant [23]. In most cases, it is not the original tumour that causes fatalities, but the spread of malignant cells to other organs. Once the primary tumour grows to a certain size, the available nutrients and oxygen become limited for the continuously dividing cells. This triggers the formation of hypoxia-inducing factors (HIFs). HIFs, with other pathways in the tumours microenvironment, trigger the expression of angiogenesis-promoting factors such as VEGF and angiopoietin-1 and 2, promoting neovascularization around the tumour [24,25]. Further, the expression of HIFs, angiogenesis, and other factors such as laminin have been shown to reduce the expression of E-cadherin through the pleiotropic effect [26]. The suppressed expression of E-cadherin in the tumour microenvironment reduces cell-to-cell adhesion and increases the mobility of the tumor cells. This begins the process of epithelial-to-mesenchymal transition (EMT). It is observed that changes in the expression of makers and molecules on its surface, such as altered expression of extracellular matrix (ECM)-cell adhesion molecules, suppress expression of epithelial markers, and increase expression of mesenchymal markers [27,28]. EMT is often considered to be the most accepted hypothesis regulating the formation of CTCs. However, this process is highly complex and still unknown. After the EMT process, the tumour cells adopt a motile, invasive phenotype leading to their detachment from the primary tumour and migration into the neighboring tissue, also known as local invasion. The process is regulated by complex mechanistic changes in cytoskeleton structure, proteolysis of surrounding tissue and alteration in cell–ECM dynamics [29]. Thereafter, some of the cancer cells evade into the network of blood vessels formed around the primary tumor body by intravasation. This process allows cancer cells to initiate metastases at a distant, different locations within the animal body. The cancer cells in the circulation (CTCs) have the ability to evade anoikis due to their mesenchymal properties and also due to the expression of anoikis inhibitors (such as XIAP) in the sub-populations of the cells, thereby resulting in the cells being resistant to apoptosis [30]. The CTCs in circulation are believed to be non-proliferative in nature and travel around the body until they adhere to the capillary bed of a different organ due to their molecular properties or size. Therefore, it is observed that secondary tumours can be formed at distant organs from the original site of the primary neoplasm causing the most fatal malignancies [31]. In addition, it is worth mentioning that these cancer cells also take part in local metastasis, thereby often reaching the lymph nodes that make the disease more severe. Once the cancer cells are attached to the capillary surface, they penetrate the endothelial layer of the blood vessel, thereby moving into the host organ. Then the cells undergo another round of phenotypic alteration almost similar to EMT which is known as mesenchymal to epithelial transition [32,33]. Though this process is less understood and is still being studied, it is observed that the cancer cells regain their epithelial characteristics, which helps them to acclimatize to new surroundings. Apart from the EMT-mediated motility of cancer cells in the circulation, the non-EMT-associated scenarios have also been known to give rise to CTCs. Centrosome amplification is one of them, in which the E-cadherin expression is maintained throughout the process of CTC formation. The centrosome is sought to play the role of organizing flagella and cilla, apart from also facilitating the separation of chromosomes. Thus, the centrosome has been shown to induce the propagation and motility of cancer cells [34]. Another method is by the non-EMT-mediated dissemination to form CTCs clusters, in which 2–50 cells are clustered together by intracellular adhesion as they move across the vessels. These cells have a higher probability of surviving anoikis than individual circulating cancer cells [35]. This contributes to a better sustainable metastasis and increases the likelihood of extravasation in secondary sites. As not all CTCs lose their epithelial properties, it is also necessary to consider these properties for the effective detection of cells in the blood. It should also be noted that cancer cells might enter the blood following the surgical or microscopic procedures to remove the tumour (such as in case of minimum residual disease) in addition to the naturally occurring CTCs from primary or secondary neoplasms [36,37].

The physical and biological characteristics of the CTCs play an important role in the development of strategies for isolation and detection. CTCs are traditionally very rare cells, and their count has been devised as the marker for the potential prognosis of cancer. Many studies have shown the presence of ≥ 5 CTCs per 7.5 mL of blood as the indication of metastatic cancer whereas one cell is considered to be the threshold for non-metastatic tumor [39,40]. However, such low cut-offs for prognosis require extremely sensitive and efficient detection methods [41]. As CTCs move around in the circulating system without any supporting tissue, these cells are found to be very fragile and harsh conditions have been shown to initiate the apoptotic cascade in them [42]. Therefore, gentle isolation methods should be followed to preserve the integrity of cells. CTCs are usually 10–20 μm, which is usually larger than the other cells in the blood. This feature is used in size-selective methods for the easy isolation of CTCs [43]. Further, the less deformability of cancer cells compared to erythrocytes and lymphocytes can be used to discriminate between malignant and non-malignant cells [44]. CTCs also contain certain biological features such as cell surface markers similar to their cells of origin. Epithelial cell adhesion molecule (EpCAM) is a transmembrane glycoprotein commonly expressed by the CTCs originating from epithelial cells [45]. The other blood cells lack this marker, thus antibodies against the molecule can be used to effectively segregate the cancer cells from the rest. However, as CTCs also undergo EMT, therefore mesenchymal markers can also be present in cells among the CTCs population. Another sub-group of CTCs is also known to show stem-cell-like features (cancer stem cells). Thus, they show stemness markers and can be used to study important aspects of cancer therapy such as recurrence and treatment resistance [46]. In addition to the common markers, cancer-specific markers are also present in CTCs. The HER2 marker has been effectively used to establish the prognosis of breast cancer CTCs [47]. In another study, androgen receptor markers have been used for the detection of hormonally responsive prostate cancer [48]. Likewise, specific markers of lung, gastric and pancreatic cancer are also used for the observation of specific CTCs.

Irrespective of the type of CTCs and how they enter the circulatory system, they undergo one the following fates: including anoikis or cell death, transition to a dormant state and remain in circulation, develop micrometastases and remain dormant for a long time without developing an aggressive tumor, or develop a malignant tumor at a secondary site [49,50]. It is believed that primary tumors can emit ~1 × 10^6^ cells/g of tumor into the circulation in a day and the majority of it (~85%) undergoes anoikis and gets destroyed within a few hours [51,52]. It is less than 0.01% of remaining cells in the circulation that act to develop metastatic tumors [36]. The rarity of the CTCs and the non-reliability of one marker to effectively distinguish them from other blood cells makes the detection and isolation of cancer cells extremely challenging. In this aspect, different markers involving liquid biopsy have been demonstrated to have significant advantages towards effective cancer monitoring (see Figure 1). The detection could involve direct or indirect methods for the identification of markers whereas isolation indicates the efficient separation of them. As CTCs are usually present in very small numbers in blood, it is necessary to develop techniques which are highly efficient, and effective as well as detectable in a small quantity of sample. After isolation, throughput methods are obligatory for the proper characterization of the isolated CTCs.

## 3. Nanotechnology

Nanotechnology has been utilized in various biological research to carry out with high-throughput, sensitivity, selectivity, and specificity and it possesses a high capacity to measure multiplexed systems. Due to their smaller size, they possess a high surface-to-volume ratio, which increases their efficiency towards cellular binding capacity; they are utilized in cancer research which has led to the advancement in significant detection, isolation and enrichment of CTCs from the blood sample. The nanoparticles that are implemented in the analysis of CTCs are classified into various types depending upon their type, shape, and structure. Some of them include quantum dots/fluorescent nanoparticles, surface-enhanced Raman scattering nanoparticles and many more. Each of them has its unique advantages and disadvantages, as discussed in Table 1, which make them suitable for cancer research. The section further explores each of the nanomaterials in detail.

### 3.1. Quantum Dots (QDs)/Fluorescent Nanoparticles

**Figure 3:**
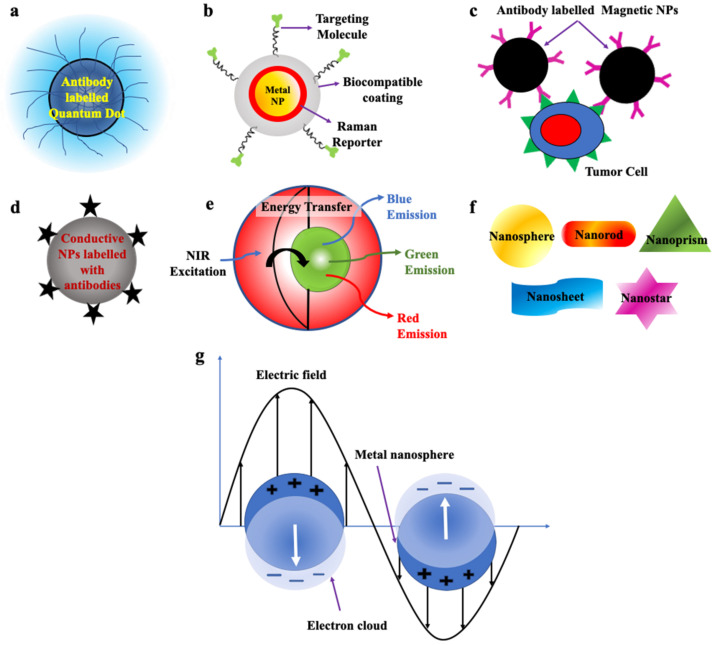
Schematic representation of (**a**) Quantum dot labelled with antibodies (Redrawn after [72]). (**b**) SERS nanoparticles (Redrawn after [73]). (**c**) Magnetic Nanoparticles labelled with antibodies (Redrawn after [62]). (**d**) Conductive nanoparticles. (**e**) Upconversion nanoparticles (Redrawn after [74]). (**f**) Nanoparticles of various morphologies used to detect CTCs. (**g**) Metallic nanoparticle displaying surface plasmon resonance (Redrawn after [75]).

QDs are zero-dimensional fluorescent nanoparticles, where all the dimensions of the particles are less than 100 nm. They possess a semiconductor core covered by a shell as shown in Figure 3a. They are generally made up of elements from groups II to VI or III to V of the periodic table. They pose discrete energy levels with varying bandgap depending upon their size, which ranges between 2–10 nm, which is less than the size of the exciton Bohr radius. They display unique electronic and optical properties such as higher quantum yield, longer fluorescence lifetime, large absorption coefficient, high-quality brightness, excellent photostability, and narrow and tunable fluorescence emission which lies between visible to infrared wavelengths. Due to these properties, QDs have led to the development of modern-day probes which have high specificity and sensitivity [53,54,55,56,71].

QDs attached to antibodies, are used in the detection of CTCs with very high metastatic potential as they display unique fluorescence signals at different wavelengths and thus, can also determine the stage of tumor by determining the number of CTCs in the blood vessel [53,54,55,56,71,76,77,78,79,80,81,82,83,84].

### 3.2. Surface-Enhanced Raman Scattering Nanoparticles (SERS NPs)

SERS nanoparticles are formed as a result of absorbance of the analyte, e.g., organic dyes on the surface of plasmonic metal nanoparticles which results in significant enhancement of their Raman signals due to the amplification of electromagnetic field upon excitation by an external light source, which helps in detection of even single molecule (Figure 3b). Its development has led to the foundation of new-generation ultrasensitive probes with high specificity and minimized photobleaching. Probes fabricated by these NPs can be used in biomedical imaging, disease detection and diagnosis [55,85].

By conjugating it with antibodies, it can be used in enrichment, multicolour imaging and enumeration of even a single cell of CTCs [57,58,59,85,86].

### 3.3. Magnetic Nanoparticles (MNPs)

Magnetic nanoparticles (MNPs) are shown in Figure 3c. They commonly pose a core–shell structure with a magnetic core that is covered by a magnetically disordered shell. They are generally made up of elements such as cobalt (Co), iron (Fe), nickel (Ni) and ferrite oxide (Fe_3_O_4_) possessing magnetic properties. When the external magnetic field is passed through them, under saturation conditions, all the magnetic moments of the particles align along the direction of the applied field. MNPs can display ferromagnetic or superparamagnetic behavior which can be changed by altering their morphology, structure and composition, which helps in their separation. If the size of the NPs is less than 20 nm, then after removal of the external field, due to strong thermal fluctuations magnetic nanoparticles display superparamagnetic behaviour, which possesses no residual magnetization in absence of a magnetic field. Whereas, if the size of the NPs is higher than this limit then some amount of residual magnetization is present in them and they are classified as ferromagnetic NPs [55,61,62].

Their unique properties such as high cellular binding capability, outstanding stability in blood and their capacity to attach a large number of MNPs to a single cell without any aggregation make them very useful for immunomagnetic separation, enrichment and detection of CTCs from blood. These NPs bind easily to the cells and, hence, can be used for in vitro separation under the presence of an externally applied magnetic field [53,60,61,62,87,88].

### 3.4. Conductive Nanoparticles/Carbon Nanotubes (CNTs)

Carbon nanotubes (CNTs) are one-dimensional nanostructures, due to the propagation of electrons only along the axis of the nanotube. They possess a hollow cylindrical core composed of single or multiple sheets of graphite (Figure 3d). Due to the strong carbon bonds in them and their cylindrical symmetry, they possess extraordinary mechanical, unique structural, and outstanding electrical properties. Their conductivity can be varied with a change in chemical binding and structure which makes them an excellent choice for the fabrication of biological sensors. If the CNTs are made up of multi-walled graphite, their surface area and electrical conductivity increased and, thus, can be utilized in the electronic detection of CTCs by the method of real-time electrical impedance sensing. When the CNTs bind with CTCs their conductivities are decreased by around 60%. They are mainly utilized to detect CTCs which have low protein expression without going through any enrichment process [54,55,63,64,89].

### 3.5. Up Conversion Nanoparticles (UCNPs)

UCNPs made up of lanthanide ions resulted in the breakthrough discovery of next-generation fluorophores, bearing the capability to convert two or more low-energy incident photons in the near-infrared region to a single photon emission with relatively higher energy in the visible region resulting in fluorescence emission. This conversion of energy occurs via a nonlinear optical process as shown in Figure 3e. The extraordinary properties displayed by them such as strong and sharp emission spectra not only prevent damage the normal tissues but also facilitate deep penetration [55,66]. Their unique luminescent properties, large Stokes shifts, low background signals, and less photobleaching, are used for high-sensitivity detection and imaging of CTCs [54,55,65,66,67].

### 3.6. Nanostructures of Various Shapes

The shape and size of the nanostructural probes greatly influence their properties and thus, their sensitivity. The most popularly utilised shapes of the nanostructure substrates are nanosphere, nanorod, nanosheet, nanoprism and nanostars (Figure 3f) [54,67,90]. Some properties associated with these nanostructures are enhanced cell capture and binding affinity which enables them to functionalise targeting ligands to be utilised for capturing and enrichment of CTCs by ligand–antigen binding. The shape and size of the NPs also affect the enhanced permeability and retention effect (EPR) [54,67,90,91,92,93,94,95].

### 3.7. Metallic Nanoparticles/Surface Plasmonic Nanoparticles

Metallic NPs display unique optical properties, due to the presence of free conduction band electrons which oscillate collectively when the frequency of the external electromagnetic radiation matches the frequency of oscillation of the conduction band electrons. Under this condition, localized surface plasmon resonance (LSPR) occurs, which results in significant enhancement of absorbance in smaller-sized metallic nanoparticles. (Figure 3g). The resonance condition in each metallic nanoparticle is dependent on its size, morphology, composition, and geometry. Plasmonic NPs such as gold and silver display biocompatibility and high chemical stability with minimum toxicity and hence, can be used for biological purposes [54,70,96].

Colorimetric assays are based on the use of metallic nanoparticles can quantifiably detect the presence of CTCs in blood. The sensitivity of functionalized metallic nanoparticles in the detection of CTCs extends up to several CTCs per mL of blood [54,68,69,97,98].

## 4. Investigation of CTCs with Nanomaterial-Based Technologies and Implementation as a Potential Biomarker for Cancer Diagnosis

Analysis of CTCs is a major challenge due to their low abundance in the blood. Therefore, sensitive technologies are developed that can isolate and detect a considerable number of CTCs from the blood even when their concentration is very little. Currently, a paradigm shift in research is being observed that focuses on the application of nanotechnology for CTC isolation and detection. The synergy between nanotechnology and CTCs has brought unprecedented advancements in fundamental arms of medical care, namely diagnostics and therapeutics. Here, we endeavored to capture, isolate and study these cells, paying particular care to illustrate their value in detecting cancer. We organized this section based on the different methods for investigating CTCs: detection, isolation and analysis. Comprehensive summarization of the different strategies is reviewed in the section below.

### 4.1. Isolation of CTC

Nanotechnology is commonly used for the efficient isolation of CTCs. Captures of these cells using various nanomaterials have shown a wide range of applications. Nanomaterials are used in different ways for the capture of CTCs from different samples. Coating nanoparticles with antibodies increases the surface area for CTCs binding. Graphene oxide (GO) chips and silica nanoparticles (SiNP), microfluidic devices are among other ways in which nanomaterials are used for sensitive CTCs capture. A comparative summarization of the different techniques involved in CTCs isolation has been performed in Table 2. With the methods discussed in this section, we aim to devise a mechanism for isolating CTCs using NPs.

#### 4.1.1. Magnetic Nanoparticle

**Figure 4:**
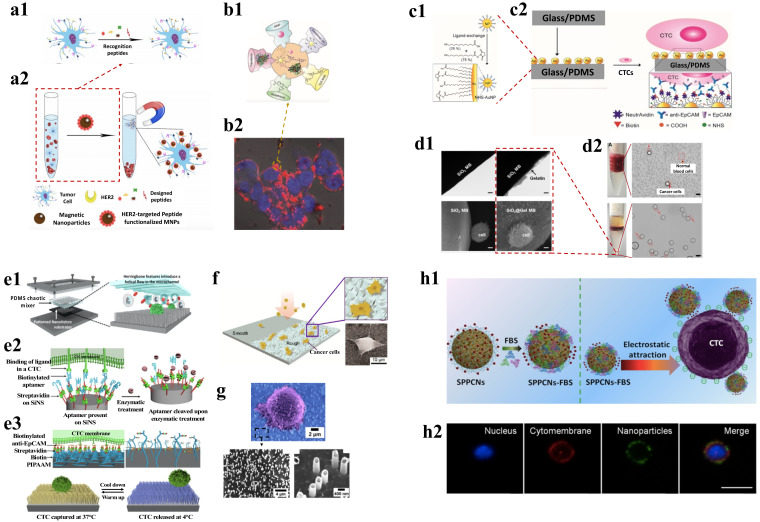
Highlights the different methods for isolation of CTCs using nanoparticles. (**a1**) Shows the schematic representation of the process that involves tagging ligand conjugated NPs to CTCs. (**a2**) highlights the capture of the targeted CTCs using an external magnet. Reprinted with permission from [120]. Copyright 2017 American Chemical Society. (**b1**) shows the different components of the MDNS whereas (**b2**) demonstrates the effective conjugation of the nanosystem on the surface of the cells (nucleus stained with DAPI appeared blue whereas MDNS appeared as red). Reprinted with permission from [101]. Copyright 2012 John Wiley and Sons. (**c1**) highlights the preparation of the functionalized NPs by ligand exchange process. (**c2**) Scheme shows the process of capturing CTCs using AuNPs in Herringbone microfluidic chip. Reprinted with permission from [104]. Copyright 2017 American Chemical Society. (**d1**) The enlarged SEM image shows the efficient binding of CTCs on the surface of gelatine NPs coated silicon beads separated by Ultra-centrifugation and (**d2**) represent the microscopic images of the captured cancer by CTC-beads (top panel) and improved selection by density gradient centrifugation (bottom panel); The scale bar is 20 μm. Reprinted with permission from [106]. Copyright 2018 Ivyspring International Publisher. (**e**) Highlights the different generations of nano-velcro for efficient isolation of CTCs. (**e1**) represents the first generation in which the Si-nanowire captures the CTCs which flow through the microfluidic device. The second generation of the system as shown in the (**e2**) panel, is based on enzymatic capture and release of CTCs whereas the third generation (shown in **e3**) uses a thermoresponsive polymer to effectively capture CTCs at 37 °C but releases the cells when the temperature reaches 4 °C. Reprinted with permission from [107]. Copyright 2014 American Chemical Society. (**f**) Shows the nanoroughened glass surface along with the SEM image of cancer cells efficiently captured by it. Reprinted with permission from [122]. Copyright 2013 American Chemical Society. (**g**) Capture of CTCs using nanostraws with the enlarged SEM image of it. Reprinted with permission from [123]. Copyright 2019 American Chemical Society. (**h1**) shows the process of capturing CTCs by electrically charged NPs, (**h2**) image panel shows the efficient binding of the positively charged Poly(ethyleneimine)-functionalized Fe_3_O_4_ NPs on the outer surface of CTCs due to effect; The scale bar is 20 μm. Reprinted with permission from [114]. Copyright 2020 American Chemical Society.

Magnetic nanoparticles (MNPs) are primarily made of magnetic elements that show alignment and attraction toward the magnetic moment in presence of an external magnetic field. The magnetic response promotes the NPs to move in the direction of the applied field, causing them to separate from the solution along with other particles to which these are conjugated, thereby developing a way for separating different analytes from the solution. Magnetic separation has been used as a leading method for enriching CTCs with high capture efficiency and specificity [99]. MNPs can be either used as embedded in polymer matrix of microbeads or as conjugated NPs. NPs can efficiently bind to cells due to their small size without causing any aggregations and also can be tagged with detection probes for simultaneous isolation–detection. MNPs functionalized with anti-EpCAM antibodies are used effectively to target and separate the cells [116]. Among the commercial magnetic separation methods, the CellSearch system is one of the most used and found to have an 80% recovery of breast cancer cells [117]. However, being able to capture only EpCAM positive cells comes as a drawback for CellSearch as the expression of EpCAM is very heterogeneous among the different cancer cells. Thus, using two anti-body systems is an advantage. Taking this forward, Wang et al., used a dual antibody system comprising anti-EpCAM and anti-N-cadherin tagged to fluorescence labeled MNPs to isolate epithelial CTCs and mesenchymal CTCs from blood samples [118]. As an EpCAM antibody independent detection method, Tannic acid functionalized MNPs were observed to have a unique interaction with the glycocalyx of the cancer cells and demonstrated to capture seven different types of CTCs (HeLa, PC-3, T24, MAD-MB-231, MCF-7, HT1080, A549) with a sensitivity of 62.3–93.7% [119]. Similarly, Bai et al. discovered that using iron oxide MNPs with the EpCAM recognition peptide as shown in Figure 4(a1,a2), enabled CTC isolation with purity above 93% [100]. Other markers recognizing peptides against HER-2 or mesenchymal can also be used in conjugation with MNPs for recognizing cells and are found to have high therapeutic potential [120]. Further, a study reported that magneto-dendritic nano systems (MDNS) with multifunction were used for rapid isolation, specific targeting, and high-resolution imaging. The components of the MDNS include: (i) transferrin, which acts as a bio-ligand to capture different subpopulations of cancer cells, (ii) iron oxide (Fe_3_O_4_), a superparamagnetic nanoparticle that helps in the isolation of cells captured using transferrin and in multimodal imaging by acting as the contrast agent, (iii) cyanine 5 NHS (Cy5) dye, a near-infrared probe used for high-resolution imaging of CTCs, (iv) fourth-generation (G4) a dendrimer having 64 binding sites for the efficient binding of functional groups such as Tf, Fe_3_O_4_ NPs, and Cy5 simultaneously and dispersibly, and (v) glutathione (GSH) functions as a reactive linker. This nanosystem effectively interacts with the CTCs and binds to its surface as shown in Figure 4(b1,b2) and allows rapid capture of the cells in less than 5 min with an efficiency of ~80% [101]. Similarly, aptamer-conjugated MPNs were also used for the isolation of CTCs. Aptamers are short strands of DNA or RNA which can bind very selectively to any specific target. Chen et al. used this strategy to selectively isolate MDA-MB-231 cells with high purity. It solved an issue of non-specific cellular uptake of peptide-MNP conjugates, as an aptamer device contains multiple copies of double-sided tape-like structures helping to effectively bind to the CTC surface [121]. Therefore, MNPs conjugated with different ligands can effectively capture the CTCs in a much simpler way and in less time when compared to other methods. However, most of the magnetic separation methods use an external magnetic field to trap the cells to the sidewall of the column. The efficiency of such methods is minimized as the magnets do not come in direct contact with the NPs. To overcome this, MagSweeper uses neodymium magnetic rods to which tumor-cells-specific antibodies such as anti-EpCAM are linked and placed directly in contact with the sample solution. As multiple such rods can come in contact with the sample, it can prevent the non-specific binding of cells as well as increase the isolation efficiency to 108 folds with 100% purity [102]. However, a drawback of MNP-based separation is that reduced or non-expression of target markers in the cells, makes it difficult to capture them.

#### 4.1.2. Non-Magnetic Nanoparticles

Many other NPs such as gold (AuNPs) and Silicon-dioxide (SiO_2_) have also shown efficiency in CTC isolation due to their ability to get conjugated with ligands and effectively bind to cancer cells. The nanoparticle CTC chip allows the capture of cells with a chemical ligand-exchange reaction (gold nanoparticle (AuNP)-thiol exchange reaction). The presence of herringbone-shaped surface ridges disrupts the laminar flow of fluid in the micro-channels, increasing the interaction between the flowing cancer cells and the nanoparticles. This technique is advantageous due to the ease of fabrication, ability to isolate CTCs with high purity, and the release of cells for downstream analyses, such as genotyping, single-cell profiling, and cytopathological analysis [103]. Similarly, Park et al. used AuNPs- thiolated ligand-exchange reaction on an HB chip for isolation of CTCs from peripheral blood. The NPs were formed from monolayers of 11-mercaptoundecanoic acid (MUA) and 12-mercaptododecanoic acid N-hydroxysuccinimide ester (NHS) and then the functionalized particles were immobilized in the chip. Figure 4(c1,c2) shows that the CTCs effectively get bound to the anti-EpCAM coated AuNPs and demonstrated a capture efficiency of 99% [104]. A thiol exchange reaction independent method was developed by Zhou et al., in which HB chips with SiO_2_ NPs are used for the isolation of cancer cells. The SiO_2_ NPs coated with anti-CD71 antibodies were able to detect wide numbers of CTCs with a capture efficiency of 16% more compared to normal HB chips [124].

A microfluidic chip coated with aptamer functionalized AuNPs was used for the isolation of CTCs in prostate and colorectal cancer patients and was found to achieve 80% efficiency with three times increase in isolation of CTCs due to the interaction between the aptamer and EpCAM. The aptamers (SYL3C) were thiolated and attached to the NPs which gave an octopus-like appearance, thereby increasing the cancer cell binding efficiency by 100 folds [105]. Similarly, chitosan nanoparticle surface coated with polyethylene glycol as an antifouling agent and DNA aptamer showed specific capture of rare CTCs from white blood cell samples [125]. Moreover, as an alternative approach to isolating. CTCs, and gelatine NPs coated silicon beads were used with density gradient centrifugation. The gelatine NPs increased the interaction of silicon beads with the cell filopodia which improved the overall cell capture efficiency by settling the heavier micro-beard conjugated CTCs to the bottom, as shown in Figure 4(d1,d2), as compared to the lighter blood cells such as WBCs and RBCs. In this study, Huang et al. used a dual antibody system (anti-EpCAM and anti-CD146) which increased the overall CTC purity to more than 85% [106].

#### 4.1.3. Nanostructure Substrates

Various nanostructured substrates such as nanopillars, nanowires, and nanofibers that are functionalised with target ligands, peptides, or antibodies, have demonstrated their capability to efficiently capture CTCs by establishing contact with various cell surface components. These nanostructure surfaces increase the surface area for cell–cell interactions as they have more target ligands per unit area of the base substrate compared to flat ones.

Lin and co-workers developed a NanoVelcro cell affinity assay for capturing CTCs. In NanoVelcro cell affinity assay, CTCs are immobilized with the help of nanostructured substrates coated with a capture agent. The NanoVelcro systems were of three generations (gens) performing different clinical applications. The first-generation of NanoVelcro consists of a silicon nanowire substrate (SiNS) and a mixer of microfluidics. The function of the device is to enumerate CTCs. The second generation of NanoVelcro uses laser microdissection methods where poly (lactic-co-glycolic acid) (PLGA) nanofiber covers are applied. The third generation of NanoVelcro chip consists of SiNS with thermo-responsive polymer brushes and is involved in capturing and releasing CTCs depending on the temperature, respectively. The Figure 4(e1–e3) showcases the different generations of NanoVelcro systems and studies suggest that the NanoVelcro CTC assay is found to be more than 85% efficient and can be used as a reliable source of biomarkers in prostate cancer. By using aptamers as a capture agent in place of Anti-EpCAM, the limitation of dissemination and translation of diagnosis based on CTCs can be addressed, which occurs due to low stability and higher cost of antibodies [107]. Various other structures such as nets and cages have also been envisioned for the isolation of CTCs. A water dispersible three-dimensional nanocage was developed by Hazra et al., from nano cellulose and iron oxide NPs. These magnetic nanoparticles were modified with the protein transferrin to capture CTCs, and they were coordinated to the molecular framework of the nano structure. The system had 85% efficiency in isolating cells from patient blood and could be used as a low-cost point-of-care diagnostic of cancer [126], whereas the two-dimensional nano-net fabricated from lipid-coated MNPs embedded on a GO sheet was used for separating the CTCs from whole blood. The nano-net was antibody labelled for specific targeting of cells, whereas the GO layer being flexible due to its 2D structure was able to easily morph around the cancer cells. Such systems overcome the drawback in most single-point cell attachment thereby providing a stable system to target cells as well as preventing non-specific attachment of cells [108].

Apart from using nanostructures, the creation of rough surfaces has been shown to increase to capture of CTC with high efficiency. Chen et al., reported a nanoroughened surface that utilised the differential adhesion preference of cells to capture them in a label-free, size-independent manner. This overcomes the issue faced due to the heterogenous expression of surface markers [127]. The Chen group further went ahead to develop a nanoroughened glass substrate that showed efficient capture of different breast cancer cells as depicted in Figure 4f, and having an efficiency of 80% when the surface roughness was 150 nm [122]. Graphene oxide (GO) nanosheets were assembled on the gold-silicon surface and was used to isolate CTCs. GO was also shown to function as a biosensor due to its biocompatibility and hydrophilicity. The surface of GO consists of many functional groups such as epoxy, carboxy, and hydroxy that can easily bind to the targets because of oxygen abundance [109].

Studies have shown that NPs conjugated with ligands can increase cell binding specificity and efficiency to a great extent. Wang et al. combined two different approaches in which MNPs functionalised with anti-EpCAM antibodies were used to capture the CTCs and then were pulled out of blood using a magnetic Si nanowire array. This method showed ~90% capture efficiency and was used to isolate lung cancer cells from clinical samples with high efficiency [128]. Further, it has been witnessed that substrates modified with NPs increase the efficiency of cell attachment. A system that used AuNPs coated with aptamer ligands has been demonstrated by Sheng et al., which was found to have increased the ability of cell isolation efficiency to 92% compared to systems that have used only aptamers [110]. Another type of structure was ZnO nanograss, which due to its ivy-like hierarchical roughness was able to yield more than 80% of the MCF-7 and MDA-MB231 cells from the sample and also showed a high CTC recovery rate [129]. Similarly, nanostraw can also be used to isolate CTCs as highlighted in Figure 4g. He et al. developed multifunctional nanostraw with nano branches which were conjugated with antibodies to capture the cells. Further, due to their hollow structure, these nanostraws were used as nanoelectroporation devices for the targeted delivery of biomolecules into the CTCs [123].

Microfluidic devices have been used to manipulate fluid at a much lower scale, which helps in making any process they carry out more efficient and faster especially in cellular biology [130]. These devices can reduce the rolling velocity of cells, which promotes enhanced cellular attachment. Using a microfluidic device with a serpentine channel for the chaotic mixing of the cells, Wang et al., used the device with anti-EpCAM-coated silicon nanopillars for capturing CTCs. The serpentine channel induces chaotic vertical flow, which increases the contact of cells with nanopillars and showed an increase in the capture yield to more than 95% [111]. In a similar study by Xu et al., nanofibers fabricated from electrospun poly(lactic-co-glycolic) acid were functionalized using hyaluronic acid and embedded on the microfluidic device. The nanofibers exhibited specificity towards the CD44 receptors on the surface of cancer cells and demonstrated more than 80% efficiency in the capture of HeLa, A549 and MCF-7 CTC [131]. A liquid biopsy-guided drug release system (LBDR-system) in a microfluidic chip was constructed to capture CTCs selectively by modifying the magnetic nanospheres (MNs) with epithelial cell adhesion molecule (EpCAM) aptamers hybridized with cDNA in such a way that one MN recognizes the CTC and the other releases the appropriate anticancer drug. It was verified that the number of released cDNAs was related to the number of detected CTCs. This can further provide treatment to the patients according to their diagnosis. The LBDR-system is a novel technique for cancer therapy [10].

Nanostructured substrates can be used for the efficient isolation of CTCs and have been one of the most promising tools in this direction. Additionally, combined with microfluidic technology, the systems have shown to require much lesser sample volume as well as reduce contamination of the cells. However, many advanced fabrication methods required to develop these structures limit their wide usage.

#### 4.1.4. Charged Nanoparticles

Cancer cells, due to their increased glycolysis, have decreased surface zeta potential. This property of the cancer cells has been utilized to devise techniques for their efficient isolation and detection [112]. Nanoparticles that are positively charged are found to bind more strongly and electrostatically to cancer cells. Surface-charged nanoparticles are proven to be more efficient in CTCs capturing than many other methods. Li et al. developed iron chloride NPs that were electrically charged with superparamagnetic properties. The negative charge on the cancer cells was used as a distinguishing character to target them with positively charged NPs and then isolated based on the magnetic nature of the particles. The system was able to segment both EpCAM positive and negative cells, irrespective of the surface markers, with more than 80.7% efficiency [113]. Similarly, electrically charged Fe3O4 composite NPs have shown efficient capture of four CTCs in 1 mL of blood sample showing high sensitivity and specificity of the method [132]. A separate study by Wu et al. reported on a Poly(ethyleneimine)-functionalized Fe_3_O_4_NPs surface coated with fetal bovine serum protein corona. Following incubation of cells and NPs, the CTCs were later effectively captured based on the CTCs surface charge, as shown in Figure 4(h1,h2), irrespective of the cell’s epithelial protein expression. The system was able to segregate 2–8 colorectal cancer cells per 1 mL of blood and was able to detect a diverse population of CTCs irrespective of their biomarkers or being heteroploidal [114]. Contrastingly non-cancerous cells with increased glycolysis can also be cornered by the method, which may result in increased non-specificity.

The majority of the nanotechnology-based CTC isolation technologies discussed above rely on microfluidic devices to collect tumor cells efficiently. Microfluidics systems have high-throughput when it comes to cell isolation and detection. Because of the transparent characteristic of PDMS, most microfluidic devices may be used for advanced microscopic and spectroscopic examination of circulating cells. Aside from nanotechnology, several approaches that manipulate fluids to build traps to isolate CTCs are being explored. Using different sheath flows or acoustic impedance to separate cells are just two of the numerous methods that have been employed [12]. One may look into many literature sources [115] for more details.

### 4.2. Probing/Detection Technologies Used in CTC

Recent studies have revealed that CTCs remain undetected in more than one-third of cancer patients because of EMT. However, a significant increase in CTC probing procedures has been seen in the last decade, and one can currently find a plethora of probing strategies for detecting CTCs. There has been a lot of progress in this field, from basic plasmonic probes to nano-bio probes. Nanomaterials in the form of nanoparticles or biosensors have been used in the detection of CTCs as they promote signal amplification. The magnetic nanosystem-based probes have also become quite popular in the detection of CTCs; however, the other probing schemes possess their own merits. The different probes have been listed in Table 3, which gives a holistic understanding of their usability. Different methods are discussed in this section, that aim to devise the mechanism of using NPs for the detection and probing of CTCs.

#### 4.2.1. Fluorescence-Based Detection Probe

Fluorescence measurement is one of the common techniques used for the detection and analysis of CTCs. Different fluorescently active molecules are used to tag the cancer cells, to detect and visualize them with high precision. Traditional organic dyes for fluorescence detection, have demonstrated low signal intensity, spectral band overlaps, and photobleaching. Overcoming these disadvantages, NPs were discovered to be more efficient. As a result, many research groups created fluorescent-based nanoprobes that are employed to identify CTCs.

**Figure 5:**
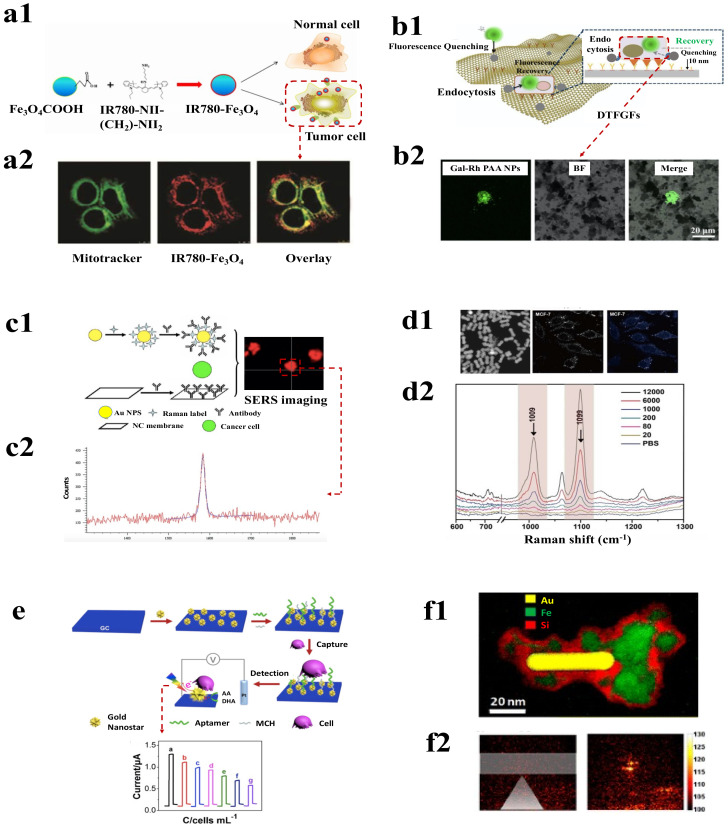
The different strategies for the detection of CTCs using NPs (part-I). (**a1**) Demonstrates the process of self-engulfing of the fluorescent magnetic IR780-Fe _3_O _4_ NPs whereas (**a2**) shows the specific localization of the NPs in the mitochondria of the CTCs. Reprinted with permission from [146]. Copyright 2017 Elsevier. (**b1**) The scheme of fluorescence detection of CTCs along with (**b2**) highlighting the fluorescence activation in the cancer cells. Reprinted with permission from [150]. Copyright 2019 American Chemical Society. (**c1**,**c2**) Shows the scheme of Au NPs functionalization followed by the capture of CTCs for SERS imaging along with the Raman spectra of a single cell. Reprinted with permission from [58]. Copyright 2014 American Chemical Society. (**d1**) Left sub-panel shows the TEM image of the nanorods whereas the middle and right sub-panels show the interaction between the nanorods and MCF-7 cells. The increase in SERS signal with the rise in cancer cell number as detected by this system has been depicted in (**d2**). Reprinted with permission from [151]. Copyright 2017 American Chemical Society. (**e**) Schematic representation of CTCs detection by the DPEE mechanism. The increase in cells concentration results in a decrease in current as detected by the potentiometer (a–g: 0, 5, 50, 1 × 10, 1 × 10, 1 × 10, 1 × 10 cells/mL respectively). Reprinted with permission from [152]. Copyright 2019 American Chemical Society. (**f1**) Shows the iron oxide NP within silica nanostructure with the three main components: Au (yellow), Fe (green) and Si (red). (**f2**) The positions of the capillary and permanent magnet are indicated in the left sub-panel whereas the photoacoustic signal of labelled HeLa cells accumulated in the detection area in the right sub-panel. Reprinted with permission from [153]. Copyright 2018 Springer Nature.

An immunomagnetic method combined with fluorescence sensing for detecting CTCs was shown by Chang et al. which utilizes magnetic mesoporous silica nanoparticles (M-MSNs) as a candidate. Fluorescent M-MSNs were turned into rod and sphere-shaped without changing their fluorescence and surface uniformity. Conjugating these M-MSNs with EpCAM enabled the enrichment and detection of CTCs based on fluorescence. This work presented how changing the shape of magnetic–fluorescent nanoprobes could contribute to the better detection of CTCs [143]. Similarly, Smith et al. have designed aptamer-conjugated magnetic and fluorescent nanoparticles, which can also simultaneously isolate and detect different types of CTCs from a single sample [144]. UCNPs fluorescence-based nanoprobes were developed for the detection of CTC by using NaYF4 (Yb: Er) UCNPs and coating them with aptamer. The limit of detection (LOD) of the nanoprobes was found to be 10 cells/10 mL of blood [145]. Exploring the self-engulfing ability of cancer cells to take up NPs, fluorescent magnetic IR780-Fe3O4 NPs were used to distinguish between normal and tumor cells, as shown in Figure 5(a1). The NPs were effectively localised in the mitochondria of the CTCs (Figure 5(a2)) which helped to be fluorescently active and provided effective localization of cells with high efficiency [146]. The ability of near-infrared (NIR) light to penetrate deep into the tissue makes it an amazing choice for biomedical imaging. Moreover, being interfered with by biological tissues also enables easy observance of different probes which emit NIR radiation [147]. Using the benefits of NIR radiations, Ding et al., developed Ag_2_S nanoprobes which are capable of emitting NIR fluorescence (at 795 nm). The probes were synthetised using the hybridization reaction from aptamer-modified Ag _2_S nanodots and were bound to immune-magnetic spheres. The system showed high efficiency in CTC capture and was able to achieve capture efficiency of six tumor cells per mL of mimicked blood sample [148]. A one-step flow-cytometry-based CTC detection approach for fluorescent silica NPs was developed by Kim et al. They developed an MNP-SiO2(RITC) system that contained organic dyes (RITC (ex/em) = 543/580 nm) and conjugated it with mucin one cell surface-associated antibodies for detecting cells. The system was able to detect 100 ovarian cancer cells in 50 μL and demonstrated high efficiency along with reducing the time of detection [149].

Multifunctional red fluorescent magneto-GCD nanoprobes were also developed by the same group which used gold nanoclusters dots (GCDs) equivalent to fermi wavelength, displaying photoluminescence properties. The nanoprobes were functionalized with cancer cell stem (CSC) markers (anti-CD34 antibody), which were used to capture and separate CSC bone marrow CD34+ stem cells from spiked blood and displayed red colour fluorescence image for excitation at 380 nm [154]. Further, this group developed a green fluorescent magneto-carbon dot (CD) multifunctional probe attached to mesenchymal markers (anti-twist antibody) to capture CAL-120 breast cancer cells that possess high levels of mesenchymal markers, and, thus, display green colour fluorescence image for excitation at 380 nm. These nanoprobes display excellent biocompatibility and photostability with QY of 0.23, with a huge amount of photoluminescence due to the presence of energy traps on the surface, which can be varied by changing the intrinsic and surface structure of the chemical groups [133,154]. The strong cell-binding capability of graphene along with its fluorescence quenching ability makes it very appealing for CTC capture and detection [155]. Wu et al. utilised this property of graphene and developed a functionalised graphene oxide film with dual targeting capability for the identification of hepatocellular carcinoma cells. The graphene surface was modified with Anti-EpCAM antibodies and galactose-rhodamine-polyacrylamide nanoparticles which initially quenched the fluorescence. However, on the binding of cancer cells, rhodamine fluorescence was recovered and could be used to visualised cells as shown in Figure 5(b2) and detected as low as five CTCs in 1 mL blood sample [150].

QDs have emerged as an excellent choice of material for biomedical imaging [156]. It can have different fluorescence illumination colors based on the size of NPs and has been effectively used for the detection of CTCs [56]. An example is using multi-enzyme NPs and quantum dots to detect cells. A fluorescent-based QD-streptavidin nanoprobe which displayed bright fluorescence, excellent resistance to photodegradation, high QY and broad excitation spectra, was used for early diagnosis and prognosis of different types of cancer diseases associated with CTCs and in detection of rare cells. They developed the nanoprobes for efficient detection and sensitive counting of specific CTCs. The probe displayed a weak fluorescent signal with RBC but displayed strong significant fluorescence with T cells (which were used as a model of CTC), whose intensity rises with an increasing population of T cells in the blood sample [157]. Kampani et al. also designed a similar nanoprobe by conjugating human T-cell leukemia virus type 1 (biot-HTLV-1) with streptavidin-coated QDs, which can be used for qualitative and quantitative detection of the rare cancer cells [158]. A magnetic fluorescent biosensor composed of graphene quantum dots (GQDs), was functionalized to the aptamer of EpCAM of cancer cells. The nanoprobe is highly efficient and sensitive with a capture efficiency of 90% for the detection and isolation of CTCs under excitation of 340 nm and 450 nm wavelength of light [135].

The microfluidic system was used to detect the tumor DNA from the CTCs by labelling them with streptavidin-fused quantum dots to fluorescently quantify the cells. The system was found to detect as low as one colorectal CTC in blood and could be used for disease-related nucleic acid monitoring [79]. Wu et al. demonstrated a high specific and sensitive strategy for immunosensing by dual signal amplification that helps in the detection of CTCs. Graphene was used to modify the surface of the immunosensor that facilitated electron transfer and silica NPs coated with quantum dots (QD) were used as tracing tags. Sandwich-type immunoreaction takes place during capturing of CTCs and the traces were captured on the cell surface. This type of immunosensor could allow the detection of cells with good stability, accuracy, and reproducibility [159]. Xie et al. proposed a multifunctional probe for the detection of specific CTC by using QDs with Ca2+ activation possessing efficiency of 86% [160]. Min et al. also adopted a similar method to design QDs modified anti-EpCAM to detect and enumerate CTCs in the blood [161]. Both the nanoprobes displayed consistent fluorescence intensity which remains constant with the number of CTCs captured. Contrastingly, changes in fluorescence intensity with the variation in pH, temperatures, and the requirement of sample preparation may come as a drawback for this type of detection technique.

#### 4.2.2. Surface-Enhanced Raman Scattering-Based Detections

Surface-enhanced Raman scattering (SERS) is a type of Raman spectroscopic method that detects the inelastic scattered light from various analytes for their identification [162]. This type of vibrational technique uses specialized substrates that enhance the Raman signal by excitation of local surface plasmons. This results in the development of an ultrasensitive plasmon-enhanced spectroscopic technique that improves Raman spectroscopy’s intrinsic specificity and operational flexibility [163].

A study by Wang et al. showed the detection of CTCs using surface-enhanced Raman scattering nanoparticles measures targeted CTCs from white blood cells. This technique overcomes limitations such as higher time consumption, lower specificity, and sensitivity. The use of epidermal growth factors as targeting ligands with SERS nanoparticles was successfully found to detect CTCs with a range of 1–720 CTCs/mL of the whole blood sample [164]. Thus, functionalizing these SERS NPs with proteins helps in the fabrication of probes that could easily bind to the surface of CTCs by surface receptor recognition. Similarly, Wu et al. used this theory and developed a highly specific and sensitive AuNPs SERS-based assay functionalised with folic acid (FA) for the detection of various types of CTCs such as (ovarian, brain, kidney, breast, lung, cervical and nasopharyngeal cells) with a LOD of 5 CTCs in 1 mL of blood [165]. Such SERS-based assays have also been used for the detection of HeLa cells in rat blood [57,68]. Further, Wu et al. developed SERS-active AuNPs of different morphologies to detect CTCs with higher sensitivity.

In a study, Zhang et al. demonstrated a noble SERS nanoprobe with NPs in triangular pyramidal-DNA. The nanostructure had strong electromagnetic hotspots at the junctions of Au NPs, that enhanced SERS signals. Further, combining the probes with antibody-conjugated aptamers showed increased efficiency in targeting the cancer cells [140]. Another interesting outcome in the detection of CTCs using SERS was the development of a system that used p-mercaptobenzoic acid molecules as SERS probes along with CTC capture substrate based on nitrocellulose membrane and PMMA wafer, as depicted in the Figure 5(c2). The system was able to detect 34 out of 100 non-small-cell lung cancer cells under a simulated environment using SERS imaging [58]. An alternative approach was used by Carmicheal et al., to detect pancreatic cancer cells based on the principle of identification of exosomes, for which positively charged plasmonic AuNPs were deposited onto negatively charged exosomes from pancreatic cancer cells. SERS spectra from the samples were collected to determine the presence of cancerous cells [166]. Park et al. also used a similar method in which they used SERS-PCA for the detection of lung cancer cells [69,167].

In addition, CTCs count in the blood can be measured by in situ monitoring of in vivo targeting using gold NPs and ex vivo detection of CTC can be performed using targeting ligands with functionalized nanoparticles. The CTC detection ex vivo is advantageous due to the potential post-capture analysis after cell culture and no toxicity to the patients. Kamińska et al. used a polymer mat with a thin layer of Ag–Au served as the SERS substrate for the effective differentiation of different cancer cells. They used multivariant analysis on the SERS data and found the 723 cm^−1^ (tryptophan) and 1452 cm^−1^ (nucleic acid nucleotides) peaks to be the most discriminating among the samples and were able to discriminate between prostate cancer, cervical carcinoma, and leucocyte cells by 95% using 2D PCA [168]. Noble gold (Au) nanomaterials such as nanoflowers and nanostars with surface modification were developed by Wang et al., for the detection of CTCs. The nanoflower and nanostar surfaces have localized surface plasmon resonance that was found to increase the SERS signals and detected a minimum of 5 cells/mL of sample [169].

The morphology of the nanostructures plays a significant role in determining the electromagnetic enhancement; highly asymmetric nanostructures possess more LSPR bands than symmetric ones. Utilising this property, Xiaoxia Wu et al. designed SERS nanoprobes by modifying AuNPs with a Raman reporter molecule, reductive bovine serum albumin (rBSA) was used to provide stability and FA was utilised to reduce nonspecific catching of healthy cells in the blood. To verify the efficiency of the probe towards detection of CTCs HepG2 cells were considered as negative cells and HeLa cells were taken to be the positive cells in a rabbit blood sample as only the later cells can overexpress FRα and thus, high-intensity SERS peak was obtained for them. These nanoprobes were used for the detection and quantitative analysis of CTCs with excellent specificity along with a low limit of detection [94,142,170]. Xiaoru Zhang et al. fabricated a triangular-pyramid-shaped DNA-AuNPs nanoprobe which displayed strong electromagnetic plasmonic hot spots between the junctions of AuNPs and resulted in the enhancement of Raman scattering. The nanoprobes helped in the highly selective and sensitive detection of (MCF-7) CTCs by the use of EpCAM aptamer. It was observed that the SERS intensity displayed by the nanoprobe is around 7.6 times greater than AuNPs−DTN, because in comparison with the plasmonic hot spots possessed by a single AuNPs, the nanogaps present in the pyramids structure of the nanoprobe provide a very high electric field intensity. Thus, MCF-7 CTCs could easily be detected at a single cell level without undergoing any enrichment process [140]. Another SERS probe was designed by Huimin Ruan et al. by modifying superparamagnetic iron oxide nanoparticles (SPION) with rBSA and FA for the successful recognition and supersensitive quantitative analysis of CTCs such as ovarian cancer, kidney cancer, breast cancer, lung cancer, etc. The nanoprisms structure shows strong electromagnetic enhancement and the MBA3-AgNPR-rBSA4-FA2 and SPION-rBSA-FA nanoparticles were used for the capture of CTCs via interaction between FA and FR α; enrichment of CTCs was performed by the use of magnetic nanosystem and detection of CTCs from the blood sample by the use of SERS phenomenon [137].

Controlling the shape, size, and number of nanogaps in the nanostructures of plasmon is vitally important while developing a quantitative sensing technology such as SERS and quantum plasmonic devices. In this work, Zhang et al. introduced coordination interactions and galvanic replacement-based synthetic methods to create core–shell plasmonic nanorods having enclosed tunable nanogaps (Figure 5(d1,d2)). Nanorods were then conjugated with MCF-7-specific aptamers. The system detected as low as 20 cells/mL of blood using SERS. Core–shell plasmonic nanorods consisting of DNA duplex could also intercalate to hydrophobic drugs; hence, they could also be used for drug delivery [151]. Recently, a microfluidics integrated system was demonstrated for using shell-isolated nanoparticle-enhanced Raman spectroscopy for the detection of flowing cancer cells. The microfluidic device first separated the cells based on inertial separation and then CTCs were detected using SERS active substrate and silica-coated NPs. The system had an accuracy of 89% with reduced detection time [171].

Thus, it can be concluded that SERS-based techniques have shown great efficiency in the detection of free-flowing cancer cells. However, there exist certain drawbacks to SERS-based detection techniques as they require to rely only on vibrational information, which limits the multi-dimensional characterization of the analytes [172]. A further requirement of specialized SERS surfaces and antibodies makes this technique considerably costly.

#### 4.2.3. Surface Plasmon Resonance-Based Detection

Surface plasmon resonance (SPR) is an optical technique that uses the surface plasmon resonance effect of the free electrons on the surface of thin metal particles to detect the presence of analytes. The target molecules when bound to the surface of metals lead to a change in the refractive index and production of surface plasmon polaritons and it is this variation in polarons that are detected as analytical measures [173]. The metal NPs have large surface areas with free electrons, making them an ideal material for SPR detection.

Functionalising AuNPs with different types of aptamers facilitates efficient capture of CTCs and their unique tuneable optical properties such as SPR help in the quantitative detection of CTCs with high sensitivity and low detection limit [106,174]. Mousavi et al. developed a simple two-step process for the detection of cancer cells. The cells were initially captured by an epidermal growth factor receptor antibody functionalised on iron-oxide NP, followed by trapping of the cells-NPs conjugates on the gold nanoslit film surface using anti-CD44 and this binding of cells on the nanoslit surface was detected by wavelength shift using SPR [175]. Jia et al. demonstrated the capture of MCF-7 cancer cells on the gold substrate, using human mucin-1 aptamers and then tagged with NPs, were conjugated with histidine-tagged arginine–glycine–aspartic acid peptide for enhanced SPR signal. The NP system showed a 20-fold increase in a signal which brought in a detection limit of 136 cells/mL of sample [176]. Haowen Huang et al. synthesized EDTA-based plasmonic nanosensors for the detection of CTCs. EDTA, under the illumination of light, catalyses to regulate the longitudinal plasmon wavelength (LPW) of AuNRs. This nanosensor potentially detects the target molecules along with a visual determination of the cancer cells. It was utilised for monitoring of MCF-7 CTCs by combining anti-EpCAM–coated magnetic beads for immunomagnetic separation of CTCs from the blood sample. These nanoprobes have unique advantages associated with them such as easy fabrication, low cost, and fast detection of CTCs [141]. The literature also contains some gene-based approaches for the detection of rare cells from blood [140]. One such gene-based method was reported by Lee et al., using plasmonic nanoparticles in a network-like structure, which can even detect a single mRNA; they further applied the process for the detection of BRCA1 mRNA splice variants. Thus, in the future, this method can be improved and can be used for quantitative analysis, and single-cell genetic profiling of CTCs [177].

In another study, Wang et al. demonstrated the detection of CTCs using a direct plasmon-enhanced electrochemistry (DPEE)-based ultrasensitive label-free method as represented by Figure 5e. A glassy electrode (GC) was modified with plasmonic gold nanostars (AuNSs) which were then immobilized with an aptamer probe for cancer cell capture. When the CTCs were captured, it reduced the excitation of localized surface plasmon resonance (LSPR) and influenced the efficiency of electrons to flow from AuNS and reduce the current response. Thus, molecular recognition by the aptamer and electrochemical response by plasmon made this system a very sensitive method with the ability to detect as low as five cancer cells/mL [152]. Likewise, Huang et al. used a signal-enhanced SPR system in which the CTCs tagged with cell membrane fragment–functionalized gold NPs and folic acid functionalized gold NPs which were then trapped in gold chip modified with anti-junction plakoglobin. This multi-signal process leads to the very sensitive detection of cancer cells with a limit of cells/mL [178].

A magneto-plasmonic nanosystem was developed by Ovejero et al., composed of gold nanorods and iron oxide NP within silica nanostructure (Figure 5(f1)). The silica provided structural stability and prevented inference between gold and iron oxide. These particles would easily bind to circulating tumor cells and can be localised using the magnetic property with subsequent detection efficiently over time using the plasmonic property by the photoacoustic method, as shown in Figure 5(f2) [153]. Detecting the DNA mutation in cancer cells is an essential strategy for the effective identification of the cancer type. Most of the time, polymerase chain reaction-based targeted gene amplification or DNA sequencing methods are employed to identify such mutations. However, performing these methods requires good amount of expertise and time [179]. Tadimety et al. developed an alternative point mutation detection strategy that used SPR-exhibiting gold nanorods conjugated with peptide nucleic acid probes complementary to the G12V mutation of the KRAS gene. The localised SPR signal was found to vary with the change in the level of mutated DNA in the sample and the system effectively demonstrated the detection limit of 2 nanograms of mutated DNA per mL in spiked samples [180].

SRP has been demonstrated to be a quick, sensitive technique without many samples pre-processing for effective detection of CTCs. However, the increase in background refractive index SPR substrate due to cross-reactivity is a downfall for its real-time applications [181]. Furthermore, SPR instruments are large and expensive which has also been a limiting factor in the exploration of the technology by the masses.

#### 4.2.4. Magnetism-Based Detection

**Figure 6:**
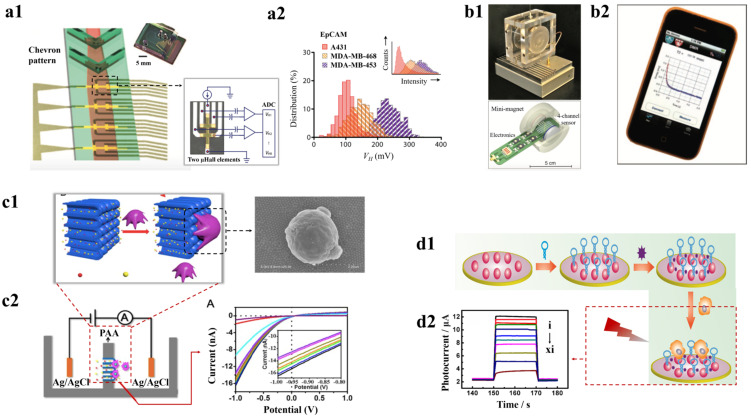
The different strategies for the detection of CTCs using NPs (part-II). (**a1**) shows the micro-Hall detector integrated with the microfluidic device. The zoomed image represents the micro-Hall element that measures the Hall voltages using capacitively coupled amplifiers, which block the constant signal from the external field and then digitized with an analog-to-digital converter. (**a2**) highlights the ability of the device to differentiate the three human cancer cell lines (A431, MDA-MB-468, MDA-MB-453). Reprinted with permission from [184]. Copyright 2012 American Association for the Advancement of Science. (**b1**) Represents the μNMR device along with its state-of-the-art μNMR probe used for sensing within the mini magnet (bottom sub-panel). (**b2**) shows the smartphone interface for operating the μNMR system. Reprinted with permission from [185]. Copyright 2011 American Association for the Advancement of Science. (**c1**) Schematic representation along with the SEM image of binding cancer cells to the nano ion-channel array. (**c2**) Shows the detection setup that detects the I-V properties of nanochannel-ion channel after capturing different concentrations of CCRF-CEM cells. Reprinted with permission from [186]. Copyright 2017 American Chemical Society. (**d1**) Illustration representing the red light-driven CTC sensing platform with the inset graphs showing the photocurrent response of the sensor toward CCRF-CEM cells. (**d2**) shows the photocurrent response of the sensor to different concentrations of cells (i–xi) 0, 1.5 × 10, 3 × 10, 6 × 10, 1.5 × 10, 3 × 10, 6 × 10, 3 × 10, 6 × 10, 3 × 10, 6 × 10 cells/mL, respectively. Reprinted with permission from [187]. Copyright 2019 Elsevier.

MNPs have demonstrated their effectiveness in the efficient isolation of CTCs. Apart from being used for isolation, MNPs have also been employed for the detection of cancer cells. Various techniques have been used to measure the magnetic properties of these cell-bound MNPs for identification purposes. A microchip-based immunomagnetic microfluidic device for probing CTCs was developed by Hoshino et al., in which at first the specific cells are labelled with an EpCAM antibody, then they are functionalized with magnetic carriers. When the blood sample flows through arrayed magnets, these CTCs which were labelled with MNPs are separated from blood and deposited at the bottom on a glass coverslip containing polydimethylsiloxane (PDMS)-based microchannel. These captured cells were then stained with fluorescence dyes followed by microscopic analysis for successful detection of very low concentrations of tumor cells in blood. The microchip’s unique flat design along with the sharp magnetic field gradient leads to significant cell capture [138]. However, this method was time-consuming and requires additional steps for CTCs enumeration. Alternatively, Chen et al. developed a rapid immunomagnetic detection of CTCs using a micromagnet array integrated into a microchip created using an inkjet printing technique. When this array is put in an external field, it enables very sensitive and selective detection of CTCs, with a 95.6% capture rate [182]. Although such technologies are capable of efficient detection of the rare tumor cells, other cell types in the blood possessing similar molecular biomarkers can pose a downfall. A promising strategy to overcome these challenges is measuring the voltage generated across the magnetic particle in the presence of an external magnetic field using the Hall effect [183]. Issadore et al. demonstrated the use of a microfluidic chip-based micro-Hall detector for detecting the Hall voltage induced by MNPs tagged cancer cells in presence of an external magnetic field as shown in Figure 6(a1,a2). As this voltage is directly proportional to the number of magnetic particles, as a result, it also detected the number of cells. The system showed a high-throughput screening ability of 107 cells/minute and showed high specificity of 96% in detecting different types of cancer cells based on the antibody conjugation in the MNPs [184]. However, NPs with weak magnetic moments are not suitable to detect cells with low expression of biomarkers.

Another approach that also has been explored to detect CTCs using MNPs is using the nuclear magnetic resonance (NMR) technique. Ghazani et al. developed a μNMR-based method that measures the water proton’s transverse relaxation time (T2) to detect cancer cells. Figure 6(b1,b2) shows the μNMR device along with its sensing probe and mini magnet. The MNPs, when tagged to CTCs, produce strong spatial-dependent local dipole fields that increase the T2 in the sample and, in turn, detect the presence of cells. The method had sensitivity and specificity of 91.6% and 100%, respectively, in detecting malignant cells and showed well differentiation between normal and cancer cells [188]. It has been observed that the MNPs with high transverse relaxivity could increase the efficiency of CTC detection. In this regard, Yoon et al., developed hybrid iron MNPs with a protective ferrite shell around the iron core to prevent NP aggregations. The system showed high cellular selectivity due to its increased magnetic moment and could detect ~10 target cells in a sample with abundance of different cells [189]. Another study by Haun et al. used a “cocktail” of four markers to simultaneously label the cells and consequently coupled them to tetrazine-MNPs. This increased the sensitivity of the system and also provided the detection of different cells within the sample at the same time. The system demonstrated 96% accuracy in detecting the cancer cells with a fast detection time of 60 min [185]. Further, in an attempt to increase the sensitivity of μNMR-based detection, Lee et al. developed a system that was able to detect 10-fold higher mass using solenoidal coils wound around the capillary tubes and produced a more homogeneous magnetic field compared to the previous example. It identified two CTCs in 1 μL of unprocessed sample and could be conducted within 15 min of performing fine needle aspiration [190]. The μNMR system increased the detection efficiency of cancer cells with reduced sample volume requirement and rapid. However, these approaches primarily depended on the immunological cell capture method for identification, which might be a disadvantage given the heterogeneity of markers. It is possible to conclude that MNPs-based systems exhibited different strategies for detecting CTCs and established that it reduced total detection time.

#### 4.2.5. Electrochemical Detection

Various electrochemical approaches are employed for the detection of CTCs. The electrochemical sensors consist of multiple electrodes and these electrodes detect the alterations on their surface due to the binding of cancer cells [191]. The electrical signal from this interaction gives an estimate of the target cells and can be measured as different parameters such as a change in voltage, impedance, current, potential difference, or conductance [192]. Moreover, different biochemical ligands are usually immobilized in the electrode surface for making the process more efficient and rapid. One of the approaches for electrochemical detection is considered to be a direct approach and focuses to hold the cancer cells onto the electrodes for detection. Cao et al. developed a method that used sgc8c aptamer conjugated on the surface of ion channel arrays for the detection of acute leukaemia CCRF-CEM cells. As the cells bind to the ion channels, as shown in Figure 6(c1,c2), it blocks the flow of ions through and this fluctuation in current is recognized using a linear sweep voltammeter to detect the presence of CTCs with a limit of 200 cells per mL [186]. Similarly, Wang et al. developed an RNA aptamer-based method for the detection of CTCs. Epidermal growth factor receptor antibodies conjugated aptamers provided easy binding with cancer cells to the microelectrode and caused an increase in resistance to the ion current between the electrodes. This system facilized easy detection of cancer cells and could be easily combined with microfluidic systems [193]. Signal amplification method developed by Zhu et al. for electrochemical detection of CTCs with high sensitivity by using self-assembled AuNPs. In this method, HER2-overexpressing cells were at first captured by anti-HER2-conjugated AuNPs and followed by counting the number of CTCs in blood by the use of Ag NPs deposited on the surface of AuNPs through the method of square wave stripping voltammetry. The LOD of this mode of detection was found to be 26 cells/mL [194]. Yi et al. also developed a similar approach for the highly sensitive detection of CTCs by using AgNPs-deposited aptamer-conjugated-AuNPs [195]. Subsequently, in an attempt to increase the sensitivity of detection, Wang et al., used plasmonic gold nanostars conjugated with a modified sgc8c aptamer. Glassy carbon electrodes were used to detect the change in current due to the local plasmonic excitation of the nanostar-tagged cancer cells and were found to detect a minimum of 10 MCF-7 cells per mL [152]. Subsequently, another way to detect the cells was to record the photocurrent in the electrodes, then the cells are excited with light. Mercaptopropionic acid (MPA)-capped AgInS2 NPs, when triggered with red light, were found to generate more current compared to when CTC was tagged to the NPs as depicted by Figure 6(d1,d2). This provided a method to detect cancer cells and detected a limit of 16 cells per mL [187].

Another method to recognize cells using electrochemical detection is to sandwich the cells with reorganization ligands and then signal amplification probes to enhance signals. Peng et al. used superconductive carbon material Ketjen black modified gold electrodes conjugated with EpCAM and anti-vimentin antibodies to capture the CTCs in samples. Further Palladium–iridium–boron–phosphorus alloy-modified mesoporous nanospheres were tagged to the captured cells as signal probes to detect the alteration in signals using differential pulse voltammetry assay. The system was able to have a minimum of two cells in the spiked samples and had considerable reproducibility even after storage for 15 days [196]. Similarly, in the study, hyperbranched PdRu nanospikes coated with platinum NPs were used by Zhou et al. for electrochemical detection of free-flowing cancer cells. The DNAzyme facilitated easy binding of the cells to the structures that were further conjugated with Super P and Au NPs for signal enhancement with the sensor, demonstrating a good correlation between the current and the number of cancer cells [197].

In addition to detecting voltammetric and amperometric changes in the electrodes to quantify CTCs, additional techniques such as impedance spectroscopy, which may detect changes in electrode impedance, were used. Shen et al. used 6-mercapto-1-hexanol coated gold electrodes that were further sensitized by aptamer bounded capture probes. The impedance value gradually increased with the rise in cancer cells in the sample and could capture a minimum of 10 cells per mL with the system that could be reused eight times [198]. Further field-effect transistors-based biosensors have been used to recognize biomolecules. These sensors are found to amplify biophysical or biochemical changes due to the interaction of biomolecules and detection receptors and lead to detecting a very minute concentration of analysts in a sample. In a recent study by Chen et al., field-effect transistors integrated into microfluidic devices have been demonstrated for the detection of CTCs. The cancer cells would bind to the detector with the help of aptamers and this binding would lead to an increase in the output signal from the transistor. The system could detect the presence of even one cell with a very slight change in signal and provided a very quick detection of CTCs within 5 min [199].

Even though the electrochemical-based method showed high sensitivity in the detection of cancer cells, such as detecting even up to two cells in samples. Most of this method relies on different detection ligands for initial recognition of the cells, which may be a downfall in the detection of non-specific cells in the samples. Nanostructured interfaces using gold nanoparticles, quantum dots, microchips embedded with nanoparticles, and so on are extensively used for the isolation, detection, and characterization of CTCs. This will provide insights into cancer metastasis and its clinical management. However, due to the low quantity of CTCs detected, their value is limited. Specificity and sensitivity remain the key limitations [106]. One of the major challenges in employing nanoparticles for CTC detection is that it has to be clinically translated. This can be achieved by synthesizing high-quality nanomaterials with mandatory post-capture analysis and low viability of captured CTCs [83]. For the characterization of CTCs at the molecular level, such as using genome sequencing and gene expression analysis, the yield and purity of the sample play an important role. Further, the detection and characterization of CTCs have potential in personalized medicines and advanced systemic therapy.

### 4.3. Nanotechnology for CTC Analysis

CTC as a biomarker for liquid biopsy technique could be used to evaluate the characteristics of tumor cells which will help in the early screening of cancer in a very feasible and non-invasive way. Analyses such as cell function profiling and downstream molecular analysis can allow us to understand the mechanism of tumor progression, tumorigenesis, and cell heterogeneity, and evaluate drug resistance, and prognosis. Nanotechnology-based analyses have proved to be extremely sensitive in clinical tumor exploration. In this section, we discuss the various applications of nanotechnology-based CTC analysis.

#### 4.3.1. CTC Transcriptome Analysis

**Figure 7:**
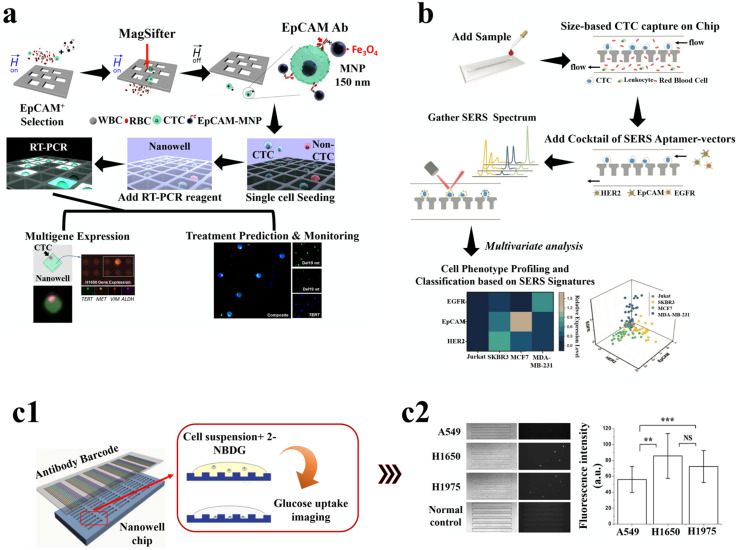
Nanomaterial-based strategies for analysis of CTCs. (**a**) shows the schematic workflow of the nanoplatform for molecular profiling of CTCs. First, the whole-blood samples are processed to isolate CTCs by the MagSifter, and biotinylated anti-EpCAM antibodies are conducted to streptavidinated 150 nm iron oxide MNPs. Followed by which the cells are loaded into nanowell device, seeded into individual compartments and mixed with RT-PCR reagent. Then the device is placed into a thermocycler for multigene expression and transcriptome analysis as well as for predicting the efficacy of cancer treatment. Reprinted with permission from [203]. Copyright 2016 Proceedings of the National Academy of Sciences of the United States of America. (**b**) demonstrates the workflow of SERS nanovectors for cell-membrane protein profiling to discriminate among the cancer cell populations. Reprinted with permission from [204]. Copyright 2018 John Wiley and Sons. (**c1**) highlights the nanowell chip-based approach for functional analysis of isolated CTCs. The cells are captured in the antibody-barcoded wells and mixed with 2-(N-(7-nitrobenz-2-oxa- 1,3-diazol-4-yl)amino)-2-deoxyglucose (2-NBDG) for glucose uptake analysis. (**c2**) shows the presence of CTCs in the nanowells as well as the representative fluorescence image showing 2-NBDG uptake. The fluorescence intensity has been shown with the bar graphs to demonstrate glucose uptake up the tumor cells (NS *p* > 0.05, ** *p* < 0.005, *** *p* < 0.0001; two-tailed Student *t* test). Reprinted with permission from [205]. Copyright 2015 American Chemical Society.

Transcriptomic analyses have widely been used for gene expression specific to tissue, disease, or cell type. However, generating gene expression profiles from single cells is quite challenging, as obtaining significant number of cells for library preparation is very critical [200,201]. However, with the use of nanotechnological solutions, it has been shown to increase the efficiency of CTC capture and isolation. CTC transcriptome analysis provided a better approach to prostate cancer (PC). Nanotechnology is now used to profile tissue-based gene expression analysis from blood. Teng et al. showed that NanoVelcro Assays could be utilized to capture and liberate CTCs having intact mRNA. RNA profiling was performed using the NanoString nCounter. The performance of PCS and PAM50 panel was examined by validating in public databases such as GnenomeDx and Prostate Cancer Transcriptome Atlas. Hence, it was proved that a contemporary tool for RNA profiling and blood component isolation was possible for genomic classification [202]. To perform single-cell RNA sequencing (RNA-Seq), parallel multigene expression profiling techniques have been developed. Park et al. reported a nanoplatform for high-throughput molecular profiling of CTCs. Compartmentalization of the nanowell device, as shown in Figure 7a, enables multiplex analysis for understanding cancer disease dynamics and provides a less invasive method for cancer assessment [203].

Cell heterogeneity could be resolved by this high-throughput single-cell RNA-Seq method from gene expression profiles and pathway regulation analysis. However, the contamination in blood and the scarcity of CTCs are the limitations of this method. The study by Cheng et al. introduced a platform for parallel single-cell RNA sequencing (scRNA-seq), Hydro-Seq. It could accommodate CTCs using its 800 chambers per chip from 10 mL blood of a patient. mRNAs from single cells are labelled by using barcodes that are paired to single cells present in the microchambers [206]., it has been found that there are various high-throughput platforms developed for single-cell transcriptomic profiling which can be used for cancer diagnosis non-invasively.

#### 4.3.2. Protein Analysis

The heterogenous molecular phenotype in-situ profiling of CTCs plays an important role in cancer diagnosis and therapy [207]. Zhang et al. proposed a combination of analysis of orthogonal multiple spectral surface-enhanced Raman spectroscopy (SERS) for cancer subpopulation identification and in-situ cell membrane protein profiling. Three orthogonal spectral aptamers of SERS were designed for phenotypic analysis, where spectral signatures were provided according to the expression of surface protein as shown in Figure 7b. The least square algorithm was used to get the phenotypic information at a resolution of a single cell by demultiplexing SERS signatures statistically. Breast cancer cell subtypes in humans were classified in combination with partial least square discriminate analysis in a highly sensitive and selective manner [204]. A study by Law et al. showed the determination of antigen concentration at a range of femtomolar by nanoparticle-enhanced biosensor integrated with immunoassay sensing technique into a surface plasmon resonance system (SPR) phase interrogation. An enhanced sensitivity was observed when plasmonic field extension was produced from the gold film to the nanorod. The antigen signal of tumor necrosis factor-alpha (TNF-α) was enhanced 40-fold with the help of gold nanorods conjugated with antibodies when compared to conventional SPR biosensing technology. Hence, gold nanorods were proved to be a potential label for amplification in SPR biosensing technology [208]. Another study by Wu et al. proposed a nanosphere system chip-assisted for the analysis of biomarker phenotype of CTCs which is performed by dual-fluorescence labelling along with magnetic tags using red and green-fluorescent bio targeting nanospheres. Magnetic enrichment allows the trapping of a single cell in a size-selective manner. Phenotypes of breast cancer cells were analysed in terms of epidermal-growth-factor receptor 2 expression level which is a target for anticancer drugs. Hence, this system could be successfully used for the detection and analysis of single-cell CTC biomarker phenotypes and could pave the way for personalized anticancer therapy [209].

#### 4.3.3. Functional Analysis

The phenotypic characteristics of CTC can be better understood by functional analysis which thereby provides a platform to study their tumor metastasis impact. The functional analysis includes metabolic characterization of properties such as cell adhesion, drug response, cell migration and the Warburg effect [210], a phenomenon where the tumor cells produce energy via glycolysis even when oxygen is present, which increases the glucose uptake. Understanding the effect of such functional parameters provides us with a better analytic impact of metastasis. Recent advancements in microfluidic and nanotechnology have led to monitoring the samples in real-time as well as simulating microenvironments, which shed light on novel profiling strategies. To determine the glucose uptake of each CTCs, a chip containing several nanoliter grooves was developed by Zhang et al., which can be assessed by fluorescent imaging of glucose analogs (Figure 7(c1,c2)). They also observed higher uptake of glucose in tumor cells with mutant EGFR compared to that with wildtype [205]. These experiments prove that the metabolism is altered by inhibiting or activating specific genes. So, another study group proposed that the metabolic activities were correlated to the expression levels of EpCAM using a microfluidic system mediated by nanoparticles [211]. The invasion mechanism of CTCs was tracked using a nano-well array that was spatially addressable. Very few CTCs showed malignancy-associated traits. Individual CTCs and those in clusters show different invasive properties [212]. The correlation of functional and molecular analysis is yet to be further explored but due to the challenging in vitro cultivation, the research is limited to cell lines.

## 5. Pharmaceutical Context

The use of nanosystems in clinical cancer management requires collaborative efforts from scientists, doctors, the pharmaceutical sector, and governing organizations. Over the past ten years, both in pharmaceutical research and the clinical setting, the use of nanotechnology to create these systems has gained widespread acceptance. Likewise, CTCs also gained substantial hype as an important marker to understand the pathology as well as the efficacy of drugs against diseases. The pharmaceutical relevance of CTCs has been highlighted with recent research showcasing the role of different factors related to CTCs to understand their adhesions at the secondary metastatic sites [213]. Organoid culture platforms generated from CTCs have been used to understand the pharmacotypic signatures related to tumors and use them as a tool for personalized therapeutics domains [214]. Researchers have also been able to study the action of drugs such as HAMPT (highly active metastasis preventing therapy) which is a combination of aspirin, lysine, mifepristone, and doxycycline against cancer cell adhesion by down-regulating integrins and CAMs, thereby reducing metastatic seeding [215]. Integration of modern pharmacological tools like OMICs and single-cell sequencing with nanotechnology for CTC analysis has demonstrated to crucially achieve longitudinal and functional characterization of the cells, providing newer insights with high-throughput and in much lesser time [216].

Further, the development of pharmaceutical nanosystems for the detection and tracking of cancer is centered on several imaging methods, including magnetic resonance, X-rays, computed tomography, positron-emission tomography, and optical imaging. They have increased both the sensitivity and specificity of detection in the spatiotemporal domain with better prediction of the treatment outcome. Drug development can be accelerated by using nanosystems coupled with therapeutics to offer relevant data on systemic toxicity, biocompatibility, pharmacokinetic, and immunogenic reactions in the body [217]. Diagnostic nanosystems have also made it to the market in some cases. Meanwhile, metallopharmaceuticals are chemicals with biological activity that includes a metal. Usually, the metal component actively contributes to the pharmacological impact that the molecule exerts. Even though metallopharmaceuticals are used in clinical settings all over the world to treat a variety of malignancies, academia continues to play a significant role in the (pre)clinical development of novel candidates. The false belief that substances containing metals are poisonous and unselective is partially to blame for this. Metal complexes may participate in redox reactions, establish covalent connections with proteins, and experience speciation in biological fluids [218]. However, we think that if these characteristics are correctly managed, they can be used in a way that allows for new activities and access to unexplored cellular signaling pathways. The absence of frequent in vivo testing is one of the biggest obstacles to the clinical development of metallopharmaceuticals. A compromise might be to put more emphasis on three-dimensional cell culture experiments, which are very inexpensive and more realistic for tumors and tissues, as these experiments can be conducted in vivo and are now cost-prohibitive in most academic settings [219].

The creation of novel anticancer medicines is still essential to eradicating the disease and reducing its unfavorable symptoms given the persistently high prevalence and death rates connected with cancer in the current day. The ongoing development of metallopharmaceuticals and other nanotechnological solutions that are clinically effective is essential for the fight against cancer. In the realm of anticancer therapies involving nanotechnology, there are still several open issues that offer new avenues for investigation.

## 6. Final Remarks and Perspectives

CTCs have been devised as an important biomarker for the detection and prognosis of cancers as well as to understand the spread over time. With the rapid development of technologies, various strategies are being used for the accurate enumeration of tumor cells. Nanotechnology has received significant attention in the last decade for the detection, isolation as well as analysis of CTCs. Owing to the unique functional and structural properties of the nanomaterials, it can aid high-throughput, sensitive, specific, and multiplexed measurement capacity at a very low cost. This review summarizes recent developments in detecting and targeting potential biomarkers, i.e., circulating tumor cells in the context of nanotechnology. Starting from the fundamentals as well as interaction mechanisms, this article presents an in-depth discussion on various aspects of CTC. With this review, we have highlighted the different nanomaterials such as metal NPs, MNPs, QDs, nano-modified surfaces, meta-NPs, or nanostructures for the effective examination of CTCs. Additionally, different techniques using NPs such as fluorescence, electrochemical, surface plasmonic resonance etc., were also discussed to understand the process employed for the effective prognosis and visualization of these cells. NPs were able to bridge current enrichment and detect methodologies to feature deeply in much of the research toward better treatments and potential cures. Analysis of the CTCs rapidly using nanotechnology has brought up an interesting niche in this area of research and can be promising for the development of better point-of-care systems for disease diagnosis. As clinical trials and patient-centric research are increasing, nanotechnology-based cancer detection methods are expected to be the first of the new technological wave to benefit the medical industry and the public in general. However, we should also be careful while using NPs-based methods for diagnosis and beware of the disposal management associated with them.

As most of the nanomaterials being exploited require surface modification, receptor sensitization as well as biological labelling, it can come as a drawback. The stability and relevant regulating required for the use of NPs can sublime the rapid growth of the field. In the future, a multidisciplinary approach should be followed for the development of nanomaterials to overcome all the current drawbacks as well as increase the sensitivity and specificity of their applications. Multi-functionalized nanoparticles are very promising as they can reduce false negatives and also could be used for multiplex detection. Development of theranostic techniques and using them with multifunctional platforms such as microfluidic devices will be an optimal strategy to look for. Another future direction of the field will be the characterization of CTCs, which may make the process of clinical decision swift and accurate as the efficiency and reliability to characterize circulating cancer cell gene and protein expression profiles remain limited. Further use of advanced computational analytical methods will be highly advantageous for successful cancer detection and treatment and will offer a multifunctional approach. As such, the use of nanotechnology for CTC analysis is a promising field that will continue to develop newer and faster technologies, bringing in changes toward better cancer treatment. Nanotechnology has shown significant potential for early detection and diagnosis of cancer. It can bring a revolution in cancer therapy in many aspects and radically increase the potency and selectivity of physical, chemical and biological approaches for identification of cancer cells. Nanotechnology uses either a passive or active approach for targeting cancer cells. Passive targeting uses the enhanced permeability and retention ability of cancer cells to increase the concentration of NPs in cells whereas molecular recognition of antigens, surface proteins, or other cancer cell surface markers are involved by active methods to localize NPs in the malignant cells [220]. Both of these approaches can be used independently or combined to enhance efficacy.

**Figure 8:**
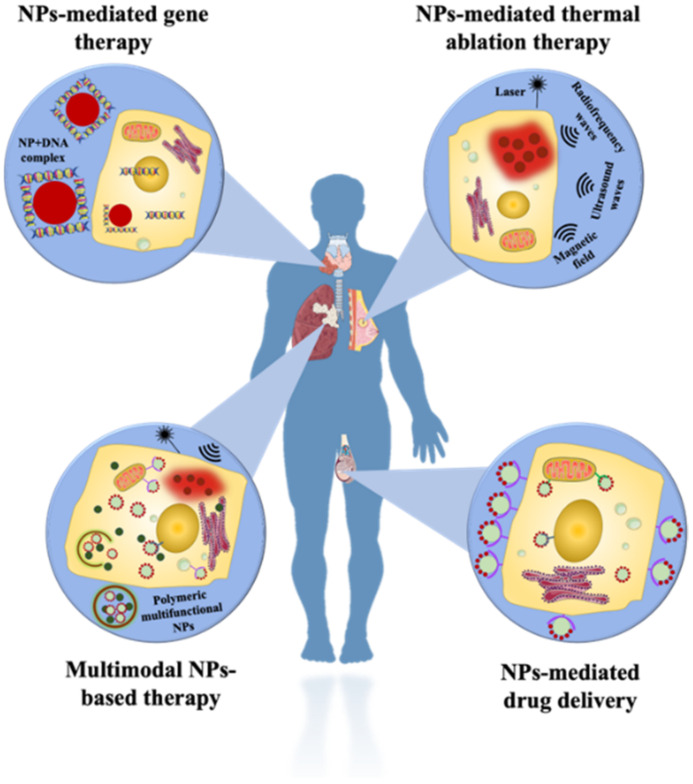
Different strategies involving nanotechnology towards treatment of cancer.

It is also worth mentioning that approaches using different NPs-mediated anti-cancer treatment strategies are being developed, as shown in Figure 8. NP-mediated drug delivery allows control release of conventional drugs depending upon the biodistribution of NPs rather than in the case of free drugs. One example is the liposome-mediated delivery of doxorubicin which has been demonstrated to significantly reduce cardiotoxicity. Further polymeric NP such as natural (albumin, chitosan, etc.) or synthetic (poly-L-lactide, poly-(L-glutamate), PEG) conjugated drugs are also exploited for therapeutics [221]. different approach uses the ability of optical or magnetic heating of NPs to locally eliminate the malignant cells. Nanomaterials such as MNPs, nanorods, carbon nanotubes, nanoshells, nanocomposites and other photodynamic materials have demonstrated significant advantages over conventional methods for thermal ablation [222]. It has been well envisioned that gene therapy shows radial advantage towards the treatment of many diseases. NPs-mediated gene therapy has been shown to enhance the targeted delivery of the genetic material inside the cells as well as significantly enhance the transfection efficiency [223]. However, the augmentation of different approaches in conjugation to each other, can enhance the effectiveness of cancer treatment. One such strategy was demonstrated with multimodal NPs, in which NPs were used for the targeted delivery of drugs as well as for thermal ablation [224]. NP-mediated therapies are highly sensitive to the treatment of cancer. Contrastingly, this field is still under development and the cytotoxic effects of materials at the nanoscale need more evaluation. Readers may find the following reviews [225,226,227,228]. to dwell deep into the topic involving NPs for cancer therapy. It is envisioned that this article will prove beneficial for researchers working in this field as well as pave the way for effective methods of probing and targeting techniques for CTCs in the future.

## Figures and Tables

**Table 1:** Different types of nanomaterials used for the detection of CTCs.

Types of Nanoparticles	Properties	Advantages	Utilization in CTC Analysis	References
Quantum Dots	Tunable narrow fluorescence emission Large absorption coefficient High brightness High photostability High quantum yield Longer fluorescence lifetime	High specificity High sensitivity	Detection of CTCs with high metastatic potential Quantified detection of CTCs in the blood vessel.	[53,54,55,56]
SERS Nanoparticle	Requires a single source of excitation Display minimized photobleaching	Helps in efficient detection of even a single cell Development of ultrasensitive probes with high specificity	Enrichment, detection, multicolour imaging and enumeration of CTCs	[55,57,58,59]
Magnetic Nanoparticle	High cellular binding capability Outstanding stability in blood Capability to attach large number of MNPs to a single cell without any aggregation.	Low cost High stability	Immunomagnetic Separation, enrichment, and detection of CTCs	[55,60,61,62]
Conductive Nanoparticles/CNTs	Extraordinary mechanical strength High electrical conductivity Conductivity varies with change in the chemical binding	Extraordinary electrical conductivity Outstanding heat conductivity	Electronic detection of CTCs with low expression of protein without any enrichment process by real-time electrical impedance sensing method	[54,55,63,64]
Upconversion Nanoparticles	Strong and sharp emission spectra High penetration depth Large Stokes shifts Low background signal High resistance to photobleaching	High photo stability High thermal stability	High sensitivity bio-detection and imaging of CTCs	[54,55,65,66,67]
Metallic nanoparticles/Plasmonic nanoparticles	Collective coherent oscillation of the electrons at resonance High amount of absorption and scattering of light at resonance	Biocompatibility High chemical stability Minimum toxicity	Colometric based detection of CTCs in blood	[54,68,69,70]

**Table 2:** Different techniques used in the isolation of CTCs.

Technique	Principle	Strategies	Advantages	Drawbacks	References
Magnetic NPs	External magnetic field causes the MNPs to move in its direction, thereby also segregating out the CTCs bound to the NPs.	Antibody and aptamer functionalised MNPs Biomolecules conjugated MNPs Iron oxide NPs Magneto-dendritic nano system etc.	Easy conjugation with ligands and CTCs Efficient and easy cell isolation and recovery process	Heterogeneous expression of surface markers makes it difficult for effective CTCs-MNPs conjugation	[99,100,101,102]
Non-magnetic NPs	NPs are surface modified to increase CTC binding for isolating them.	Ligand modified Au and Ag NPs Surface modified NPs SiO_2_ NPs etc	Reduces the dependency on surface protein markers for targeting cancer cells Increased biocompatibility Heterogenous CTCs populations can also be isolated	Production of modified NPs requires specialized techniques and expertise	[103,104,105,106]
Nano-structures	Different structures incorporated of nanoscale are incorporated to increase the surface area for cell binding	Nano velcro, nano cage, Nano wires, Nano pillars, nano grass, nano straw Nanoroughened surfaces, etc.	Enhanced local topographic interactions between the substrates and targeting cell Nanostructures can be coated with ligands with much higher densities than flat surfaces, improves binding affinity Nanostructures embedded into a microfluidic device, lower the rolling velocity of cells in microchannels channels and enhance cellular binding	Multiple fabrication methods required to develop these structures limit their wide usage Higher nonspecific binding is observed for non-functionalized nanoroughed substrates	[107,108,109,110,111]
Charged NPs	Separates CTCs based on the surface charge of the cells. The surface negative charge in cancer cells is due to high glycolysis (Warburg effect)	FeCl_3_ and Fe_3_O_4_ NPs electrically charged with superparamagnetic properties	Electrical charged NPs avoid the immobilization of antigen on magnetic beads, which overcome the limitation caused by lacking attachment The viability of isolated CTCs is not to be influenced by electronical charged nanoparticles, which is more important for further studies	Non-cancerous cells with increased glycolysis can also be cornered by the method which may result in increased non-specificity	[112,113,114,115]

**Table 3:** List of various probes based on fluorescence, magnetic, surface-enhanced Raman scattering, surface plasmon resonance-based techniques for detection of CTCs.

Type of Probe	Detection Technique	Working Principle	Advantages	Applications and Targets	References
PD-conjugated blue fluorescent−magnetic nanoprobe	Fluorescence-based detection	Magnetic separation followed by capture of the targeted CTCs. ELISA and fluorescence mapping analysis used for characterisation and imaging of CTCs.	Biocompatible Photostable Capture efficiency of 97%.	SK-BR-3 epithelial cancer cells.	[133]
GCD-conjugated red fluorescent−magnetic nanoprobe	Fluorescence-based detection			Bone marrow CD34+ stem cells.	[133]
Green fluorescent magneto-CD nanoprobes	Fluorescence-based detection			CAL-120 breast cancer cells.	[133]
DNA-templated MNP-QD-aptamer copolymers (MQAPs)	Fluorescence-based detection	Gathers amplified magnetic response. Extraordinary binding selectivity Ultra-bright ensemble of QD PL for single cell detection.	Isolation and enumeration of rare types of CTCs with high accuracy and sensitivity up to 80%.	Ultrasensitive isolation and detection of CTCs.	[134]
Fluorescent QD625–streptavidin on T cell	Fluorescence-based detection	Conjugation of CTCs with cells, where the biotin–streptavidin acts as a bridge to connect QDs and T cell.	Efficient detection Rapid detection Low cost High sensitivity.	Sensitive, efficient counting and detection of a specific rare type of CTCs cell.	[77]
Aptamer-Fe_3_O_4_-GQD-MoS_2_ nanoprobes	Fluorescence-based detection	The EpCAM separates MoS_2_ nanosheets from the nanoprobe.	High capture efficiency upto 90%.	Rapid, efficient, and sensitive separation and detection of CTCs.	[135]
Avidin-conjugated Fluorescent-magnetic biotargeting-nanoprobes	Fluorescence-based detection	The labelled cells were incubated and subjected to magnetic separation. Fluorescent microscopic images of the precipitate were obtained.	High capture efficiency upto 96%.	Early diagnosis Specific and sensitive detection Rapid separation of spiked leukemia cells and prostate cancer cells.	[136]
SPION-rBSA-FA and MBA-AgNPR-rBSA-FA nanoprobes	Surface-enhanced Raman Scattering-based detection	Rabbit blood is incubated with nanoprobe. Isolation by magnet Detection by a Raman instrument.	Supersensitive and quantitative analysis of CTCs.	Detection, capture and enrichment of ovarian cancer, kidney cancer, breast cancer and lung cancer cells.	[137]
Microchip-based immunomagnetic assay	Magnetism-based detection	CTCs were deposited at the bottom by magnetic separation, Observed under a fluorescence microscope.	Less than 25% of MNPs are required compared to CellSearch™ system.	Detection of rare cancer cells with very low tumor cell to blood cell ratios.	[138]
Magneto-Dendrimeric nano system (MDNS)	Magnetism-based detection	Fe_3_O_4_ MNPs were used for magnetic isolation Cy5 NHS was used to enable high-resolution imaging of the CTCs.	Highly efficient in isolating and targeting CTCs.	Used for simultaneous tumor cell-specific affinity, resolution confocal imaging, and cell isolation.	[139]
Triangular Pyramid (TP) DNA-Au NPs probe	Surface-enhanced Raman Scattering-based detection	The probes were coupled with the aptamer EpCAMs and mixed with the sample, then they were subjected to SERS detection.	Highly sensitive in detection of CTCs.	Detects MCF-7 CTCs at the single-cell level from human blood sample without enrichment process.	[140]
EDTA-Based Plasmonic Nanosensor	Surface plasmonic resonance based detection	Target interacts with antibodies which are recognized by secondary antibodies attached with EDTA. The presence of AuNRs and H_2_O_2_ produces colour change.	Enables naked eye observation and determination of CTCs.	Detects CTC MCF-7 with high sensitivity and accuracy.	[141]
AuNP−MBA−rBSA−FA nanoprobe	Surface-enhanced Raman Scattering-based detection	The SERS-active NPs is used to reduce nonspecific catching of healthy cells and then FA was used to recognise various types of CTCs.	Possess excellent specificity High sensitivity Cost-effective Displays strong SERS signal.	Detection and quantitative analysis of CTCs such as MCF-7 and HeLa cancer cells in rabbit blood sample.	[142]
AuNS-MBA-rBSA-FA nanoprobe			Efficient detection of CTC without going through any enrichment process.	Detection of CTCs such as HeLa cells in rabbit blood sample without any enrichment.	[94]
AuNR-MBA-rBSA-FA nanoprobe					[94]

## Data Availability

Not applicable.
